# Optimization of protocols for pre-embedding immunogold electron microscopy of neurons in cell cultures and brains

**DOI:** 10.1186/s13041-021-00799-2

**Published:** 2021-06-03

**Authors:** Jung-Hwa Tao-Cheng, Virginia Crocker, Sandra Lara Moreira, Rita Azzam

**Affiliations:** NINDS Electron Microscopy Facility, National Institute of Neurological Disorders and Stroke, National Institutes of Health, Bethesda, MD 20892 USA

**Keywords:** Synaptic proteins, Electron microscopy, Immunogold labeling

## Abstract

Immunogold labeling allows localization of proteins at the electron microscopy (EM) level of resolution, and quantification of signals. The present paper summarizes methodological issues and experiences gained from studies on the distribution of synaptic and other neuron-specific proteins in cell cultures and brain tissues via a pre-embedding method. An optimal protocol includes careful determination of a fixation condition for any particular antibody, a well-planned tissue processing procedure, and a strict evaluation of the credibility of the labeling. Here, tips and caveats on different steps of the sample preparation protocol are illustrated with examples. A good starting condition for EM-compatible fixation and permeabilization is 4% paraformaldehyde in PBS for 30 min at room temperature, followed by 30 min incubation with 0.1% saponin. An optimal condition can then be readjusted for each particular antibody. Each lot of the secondary antibody (conjugated with a 1.4 nm small gold particle) needs to be evaluated against known standards for labeling efficiency. Silver enhancement is required to make the small gold visible, and quality of the silver-enhanced signals can be affected by subsequent steps of osmium tetroxide treatment, uranyl acetate en bloc staining, and by detergent or ethanol used to clean the diamond knife for cutting thin sections. Most importantly, verification of signals requires understanding of the protein of interest in order to validate for correct localization of antibodies at expected epitopes on particular organelles, and quantification of signals needs to take into consideration the penetration gradient of reagents and clumping of secondary antibodies.

**Supplementary Information:**

The online version contains supplementary material available at 10.1186/s13041-021-00799-2.

## Introduction

Although the development of super-resolution microscopy at the light microscopy (LM) level enabled the observation of suborganellar localization of molecules [1, 2], electron microscopy (EM) still offers higher resolution images to match the molecular localization at ultrastructural level. Immunogold labeling of endogenous proteins with specific antibodies at the EM level allows localization of these proteins in intact cells [3, 4]. Information on the distribution and quantification of these proteins under different stimulation conditions can offer important clues to their functions [5, 6].

There are several approaches of EM immunolabeling, each with advantages and limitations [7–10], and none can be accomplished without much endeavor. The present paper summarizes tips and caveats of the pre-embedding technique of immunogold EM in dissociated rat hippocampal neuronal cultures and in perfusion-fixed mouse and rat brains, with particular focus on synaptic and other neuronal proteins. This pre-embedding method can be achieved by any EM laboratory with standard technique [9] without specialized low temperature equipment required for cryo-thin sectioning [11], or for rapid freezing and freeze-substitution essential for post-embedding methods [10]. Thus, this pre-embedding method is the easiest to try among the various techniques.

For optimal structural preservation, samples processed for EM require a stronger fixation and weaker permeabilization conditions than those processed for immunofluorescence LM. Thus, not all antibodies that work for immunofluorescence LM will work for immunogold EM. The present paper will show examples of assessing optimal fixation and permeabilization conditions for each particular primary antibody.

The present paper will also illustrate the importance of evaluating the labeling efficiency of each lot of secondary antibody conjugated with a small (1.4 nm) gold particle [12, 13], which will require silver or gold enhancement to become visible signals [14, 15]. Here, different enhancement reagents are compared to illustrate their specificity and efficiency. The enhanced signals can be affected by the concentration and composition of osmium tetroxide and uranyl acetate (UA) treatment during processing, and the silver particles in thin sections can deteriorate over time. The present paper lists examples of potential flaws and means to avoid them.

The present paper also lists several examples on interpretation of signals and means to verify the specificity of antibodies. We also list some common artifacts and cautions in quantifications of signals.

## Methods

### Antibodies

The primary antibodies used in present study are listed in Table 1, sorted by groups of target proteins with their expected locations in neurons.

**Table 1:** Specifics on primary antibodies

Target protein	Location in neurons	Antibody species & clone number	Sources
Synaptophysin	Synaptic vesicle (SV) membrane	Rabbit polyclonal (pAb)	DAKO (Glostrup, Denmark)
SV2	SV membrane	Mouse monoclonal (mAb), clone 10H3	gift from Dr. Erik S. Schweitzer
VGluT (vesicular glutamate transporter)	SV of glutamatergic terminals	Rabbit pAb	Synaptic Systems (Goettingen, Germany)
synapsin I	SV-associated	Mouse mAb clone 46.1	Synaptic Systems
α-Synuclein	SV-associated	Mouse mAb clone 42	BD Biosciences (San Jose, CA, USA)
Bassoon	Presynaptic active zone cytomatrix	Mouse mAb clone SAP7F407	Stressgen (Victoria, BC, Canada)
GluR2 (AMPA receptor)	Postsynaptic membrane	Mouse mAb clone 6C4	Millipore (Billerica, MA, USA)
NR2B (NMDA receptor)	Postsynaptic membrane	Mouse mAb clone N59/36	NeuroMab (Davis, CA, USA)
NR2A/B (NMDA receptor)	Postsynaptic membrane	Rabbit pAb	Chemicon (Temecula, CA, USA)
PSD95	Postsynaptic density (PSD)	Rabbit pAb raised against residues 290–307	Custom-made by New England Peptide (Gardener, MA, USA)
synGAP	PSD and cytosolic	Rabbit mAb clone EPR 2883	Millipore
pan Shank	PSD	Mouse mAb clone N23B/49	NeuroMab
Shank 2	PSD	Mouse mAb clone N23B/6,	NeuroMab
Shank 3	PSD	Rabbit pAb	Santa Cruz (Dallas, TX, USA)
Homer 1	PSD	Rabbit pAb	Synaptic Systems
Homer 1b/c	PSD	Rabbit pAb	Synaptic Systems
Homer 2	PSD	Rabbit pAb	Synaptic Systems
Homer 1/2/3	PSD	Rabbit pAb	Synaptic Systems
IRSp53 ab 1	PSD	Mouse mAb clone L117/1	NeuroMab
IRSp53 ab 2	PSD	Rabbit pAb	Protein Tech Group (Rosemont, IL, USA)
α-CaMKII	PSD and cytosolic	Mouse mAb clone 6G9(2)	Millipore
Ip3 receptor	Endoplasmic reticulum (ER) membrane	Mouse mAb clone L24/18	NeuroMab
Ryanodine receptor	ER membrane	Mouse mAb clone 34C	Affinity BioReagents (Golden, CO, USA)
Chromogranin A	Dense core of dense core granule	Rabbit pAb	gift from Dr. Lee Eiden (NIMH, Bethesda, MD, USA)

Nanogold-Fab’ secondary antibody (1.4 nm-sized gold covalently conjugated to the Fab’ fragment of IgG), the HQ silver enhancement kit and the gold enhancement kit “Goldenhance EM” were from Nanoprobes (Yaphank, NY, USA); AURION R-Gent SE-EM, a silver enhancement kit from Aurion (Wageningen, the Netherlands) was distributed by Electron Microscopy Sciences (EMS, Hatfield, PA, USA).

### Fixation of dissociated rat hippocampal cultures

Dissociated rat hippocampal neuronal cultures were grown as described [16]. Cultures were either grown on substrate-coated glass coverslips or on plastic tissue culture wares. The standard initial fixation in this lab for testing any new primary antibody is to use freshly diluted 4% paraformaldehyde (PF, 16% stock in sealed vials from EMS) in phosphate buffered saline (PBS) for 30 min at room temperature. Fixed samples were washed with PBS 4 times with 1 quick wash plus 3 times at 5 min interval each. Samples can then be stored at 4 °C if need be. A storage time for up to 1 week yielded similar immunolabeling results for many antibodies. Depending on the initial assessment of the results, the concentration of PF and timing of fixation can be adjusted. For some antibodies, a low concentration of glutaraldehyde (0.05–0.2%) can be included to achieve better structural preservation. The optimal fixation conditions will have to be determined for each particular antibody (cf. Additional information in [17, 18]).

### Perfusion fixation of adult mouse and rat brains

A good perfusion fixation [3, 19] will quickly bring fixative throughout the brain via the blood vessels, and the tissue will retain the fixative if the brain is kept intact and not cut up into thin slices or small pieces after the perfusion fixation. The standard procedure in this lab is to perfusion fix with 4% PF in PBS at room temperature, and to let the fixative stay in the brain not longer than 40 min to prevent over-fixation, which can lead to lower immunolabeling efficiency. Typically, the perfusion-fixed brain was dissected out and immersed in the same fixative until ready for vibratoming. The fixed brain was then transferred into PBS and vibratomed into 100 µm thick slices in PBS. After 3 more washes in PBS at 5 min each, the brain slices are considered cleared of fixative and ready for immunolabeling. The brain slices can be stored in PBS at 4 °C for up to 7–10 days without noticeable deterioration in structural preservation or labeling efficiency for most antibodies. The brain slices were then processed free-floating in 24-well tissue culture plates.

Immersion-fixed brain tissues, including human brain tissues removed during surgery or harvested postmortem, can also be vibratomed into 100 µm thick slices and then processed for immunolabeling. However, it should be noted that neurons in these samples are likely under excitatory conditions due to excision and a delay in fixation [19], and that structural preservation may not be as good as that from perfusion-fixed samples.

### Standard protocol of pre-embedding immunogold labeling

**Fig. 1:**
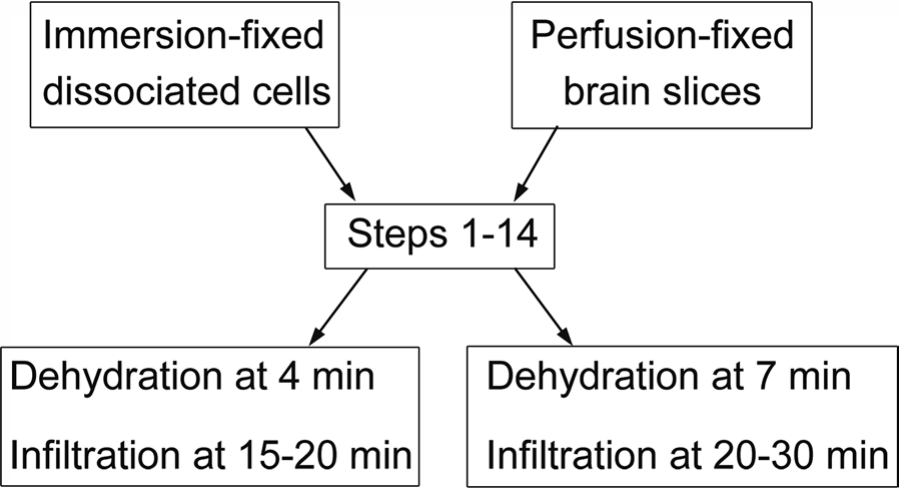
Schematic flowchart of tissue processing protocols for dissociated cells (left) and brain slices (right)

The following steps are the standard procedure in this lab for initial testing with any new antibody, and differences between dissociated cells and brain slices are listed in a flow chart (Fig. 1):

1. Block and permeabilize thoroughly-washed samples with PBS containing 5% normal goat serum (NGS) and 0.1% saponin for 30 min. Other reagents may be substituted for NGS to block non-specific labeling pending empirical results for each particular antibody.
2. Incubate with primary antibody made in PBS containing 5% NGS and 0.05% saponin for 1 h at room temp. Carrying a control sample with no primary antibody incubation is strongly recommended.
3. Wash with PBS 4 times with 1 quick wash plus 3 times at 5 min interval each.
4. Incubate with secondary antibody (Nanogold-Fab’ from Nanoprobes at 1:200) in PBS containing 5% NGS and 0.05% Saponin for 1 h at room temp.
5. Wash with PBS 4 times with 1 quick wash plus 3 times at 5 min interval each.
6. Fix with 2% glutaraldehyde in PBS for 30 min at room temp, then store in fixative in refrigerator at 4 °C if need be. A storage time for up to 4–6 weeks yielded similar immunolabeling results for many antibodies.
7. Wash with deionized water 5 times with 1 quick wash plus 4 times at 5 min interval each.
8. Silver enhance samples with the HQ kit from Nanoprobes in darkroom conditions under a red safety light. Free-floating brain slices were transferred into plastic baskets for easy handling and better control on solution changes under the safety light condition. Quickly mix equal volumes of the three reagents of the HQ silver enhancement kit and use immediately at room temperature. Optimal silver enhancement time should be determined for each antibody and for each lot of the HQ kit. Currently, a treatment time of ~ 8–12 min is the norm in our hands.
9. Wash samples with deionized water with 3 quick washes plus 5 times at 2 min each. After silver enhancement, samples typically showed a yellowish/brownish color visible by eye, and signals appeared as a dark brown reaction product under LM. The pattern of labeling under LM should be consistent with that of fluorescence images. Control sample with no primary antibody should not have visible signals and can serve as a quick verification of the validity of the immunolabeling procedure up to this step. On the other hand, if no or very little signal was detected from samples treated with primary antibody at this stage, samples can be further silver enhanced for additional time. We typically abandon samples if no signal is detected after a maximal of 15–20 min of silver enhancement. Experiments would be repeated at lower fixation conditions and/or higher concentrations of the primary antibody to increase labeling efficiency.
10. Change samples into 0.1 M phosphate buffer at pH 7.4 on ice and treat with fresh-made 0.2% osmium tetroxide [diluted from sealed vials of 4% aqueous stock (EMS)] in 0.1 M phosphate buffer at pH 7.4 for 30 min on ice.
11. Wash in 0.1 M phosphate buffer with one quick wash and 2 times at 5 min each on ice, then either treat with steps 12–14 or proceed directly to dehydration with steps 15 onward.
12. Wash in 0.1 N acetate buffer at pH 5.0 with one quick wash and 2 times at 5 min each on ice. (0.2 N acetate buffer stock was made with 0.2 N sodium acetate and 0.2 N acetic acid with a 3:1 volume ratio.)
13. Treat samples with 0.25% uranyl acetate (UA) in 0.1 N acetate buffer for 1 h on ice. The UA solution should be stored light-tight and cold at 4 °C.
14. Wash in 0.1 N acetate buffer thoroughly with 1 quick wash and 3 times at 5 min each on ice.
15. Dehydrate samples at room temperature in a graded series of ethanol at 50%, 70%, 90%, and 3 changes of 100%, and infiltrate in ethanol and epoxy resin mixtures at 1:1, and 1:2. It should be noted that cell culture samples benefit from a dehydration protocol with intervals shorter than the conventional 10 min interval for glutaraldehyde-fixed tissues. In this lab, immunogold-labeled monolayer samples were dehydrated at 4 min intervals, and perfusion-fixed brain slices were dehydrated at a 7 min schedule. This shortened dehydration schedule is helpful in preserving membrane structure for PF-fixed samples especially those that were fixed at a lower concentration for a shorter period of time. Time of infiltration with mixture of ethanol and epoxy resin varied between 15 and 30 min depending on the size of the sample. Monolayer samples were infiltrated between 15 and 20 min, and brain slices were infiltrated between 20 and 30 min.
16. Embed samples after 2 changes of 100% epoxy resin for 45–60 min in 50 °C oven, polymerize samples in the third change of resin at 50 °C overnight, and at 60 °C for 2 more days. Cell culture samples grown on tissue culture ware were embedded in the wells they were grown in, and cells grown on glass coverslips were embedded inverted, overhanging shallow embedding molds (Pelco DiscBlock embedding mold, Ted Pella, Redding, CA, USA) with the opening of the molds smaller than the size of the glass coverslips. Brain slices were either flat-embedded between two Permanox plastic slides (Thermo Fisher Scientific, Rochester, NY, USA), or with the interested area of the brain dissected out and oriented for cross-section in regular embedding molds.

### Thin sectioning and electron microscopy

Epoxy-embedded cell culture samples were separated from the glass coverslips or the plastic tissue culture ware, and sections were typically cut *en face*. Flat embedded brain slices were trimmed for interested area of the brain and then oriented for either cross or *en face* sections. For projects that need quantification of signals, brain slices were cross-sectioned, and sampling was restricted to the most superficial 1 µm of the cut edge where labeling efficiency is consistently higher than the areas deeper into the tissue.

400-mesh hexagonal copper grids were cleaned with acetone and air-dried before use, and ethanol should be avoided in cleaning grids because the residue left on the grids may interfere with silver-enhanced signals. The diamond knife used to cut thin sections should never be cleaned with detergent and should be rinsed and soaked with water overnight if it was cleaned with ethanol.

Thin sections at ~ 70 nm were stained with 1% UA for 15 min followed by 3% lead citrate for 5 min if the samples were not treated with UA en bloc during tissue processing. Samples that had UA en bloc staining can have sufficient contrast without the UA counterstaining of the thin sections. Sections were examined on a JEOL 1200EXII or a JEOL 200CX transmission EM and images were collected with a CCD digital camera system (XR-100 from AMT, Danvers, MA, USA).

## Results and discussions

Considerations and caveats at each step of the tissue processing protocol for pre-embedding immunogold labeling EM are listed here:

### Initial fixation affects structural preservation and immunolabeling efficiency

**Fig. 2:**
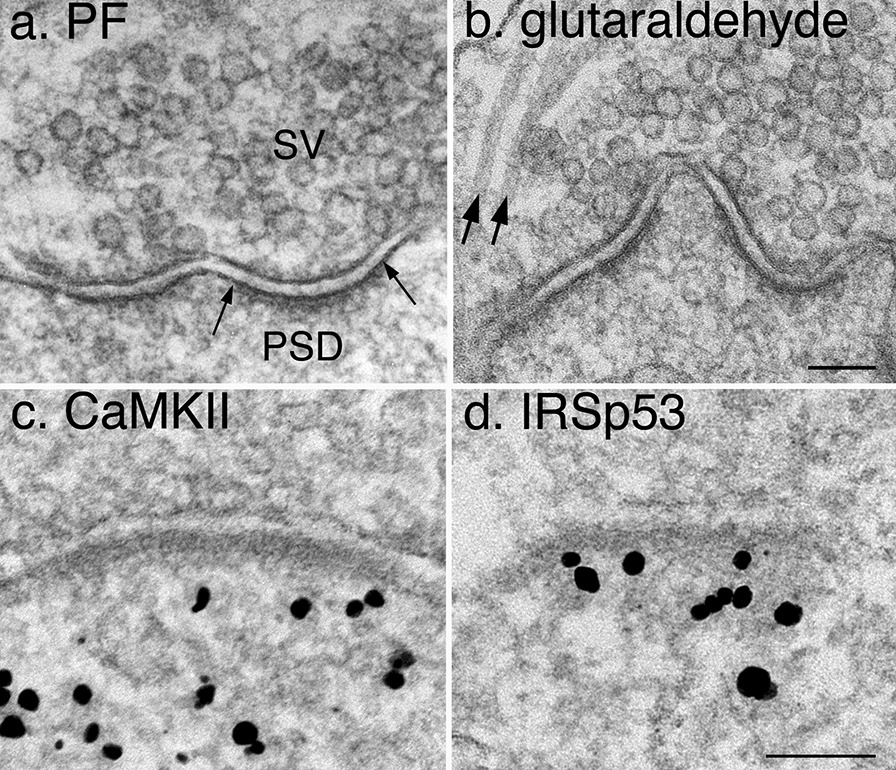
Comparison on structural preservation upon different fixation conditions and subsequent treatments. Images show synaptic profiles from 3 week-old dissociated hippocampal cultures without (**a**, **b**) or with (**c**, **d**) immunogold labeling. Clusters of synaptic vesicles (SV in **a**) and the postsynaptic density (PSD, edges of which marked with arrows in **a**) are characteristics of glutamatergic asymmetric synapses. The fixation treatments were 4% PF in PBS for 45 min (**a**), 4% glutaraldehyde in 0.1 M cacodylate buffer at pH 7.4 for 30 min (**b**), 4% PF in PBS for 30 min and labeled for CaMKII (**c**), a cytosolic protein in neurons [6], and 2% PF in PBS for 15 min and labeled for IRSp53 (**d**), an actin-associated protein enriched at the PSD [20]. All fixations were carried out at room temperature. Membranes were poorly preserved in (**d**) due to the lower concentration of PF and the shorter fixation time. Scale bars = 100 nm

Fixation typically decreases immunolabeling efficiency because the fixatives denature proteins and may alter the binding of antibodies to the epitopes of the antigen [8, 9]. In general, the classic EM fixative, glutaraldehyde, is not compatible with immunolabeling for the majority of antibodies, and paraformaldehyde (PF) is the preferred fixative for immunolabeling [8, 9]. However, contrary to conventional assumption, quality of structural preservation of PF-fixed samples can be reasonably high if samples are optimally processed. Figure 2a shows an example of dissociated hippocampal cultures fixed with 4% PF in PBS for 45 min. The membranes and various organelles were similarly well-preserved when compared to a sample fixed with 4% glutaraldehyde in 0.1 M cacodylate buffer (Fig. 2b). One conspicuous difference is that microtubules were only preserved in glutaraldehyde-fixed samples (arrows in Fig. 2b) but not in PF-fixed samples (Fig. 2a).

Notably, structural preservation in PF-fixed samples without immunogold-labeling (Fig. 2a) is of better quality than those with labeling (Fig. 2c and d) because the latter need to go through permeabilization treatment and extensive washing between the various incubation steps. Typically, labeling efficiency is in reverse proportion to the degree of fixation [8, 9]. For some antibodies, the concentration of fixative and time of fixation had to be decreased to a level where structural preservation was compromised. For example, structural preservation was worse in the sample fixed at a lower PF concentration for a shorter time (Fig. 2d) than the sample in Fig. 2c. However, even though membranes were not optimally preserved in this lesser-fixed sample (Fig. 2d), the resulting labeling at the postsynaptic compartment is still credible and quantification of labeling density is valid [20].

For perfusion-fixed brain tissues, over-fixation is a common cause for low labeling efficiency. Additional file 1a shows a brain sample that was perfusion-fixed with 4% PF for 60 min before the brain was vibratomed into 100 µm slices and washed in buffer. The labeling intensity with a Shank 3 [21] antibody at the postsynaptic density (arrows in Additional file 1a) was lower than that in another sample that was perfusion-fixed for 40 min (Additional file 1b).

### Permeabilization conditions affects membrane structure and labeling efficiency of plasma membrane proteins

Immunolabeling reagents often need to pass the biological membranes to reach their target epitopes. A common reagent to make the membranes permeable is detergent, which modifies the membranes to different degrees depending on the type of detergent, and its concentration and treatment time. The typical permeabilization condition we chose for the first try with any antibody is a 30 min incubation in 0.1% saponin at room temperature. With this treatment, the structure of the biological membrane was generally acceptably preserved even with a very mild fixation at 15 min of 4% PF.

Saponin works preferentially on cholesterol, which is more abundant in plasma membranes (PM) than in other biological membranes [22], and thus, may selectively alter the configuration of epitopes of PM proteins. In turn, labeling efficiency of antibodies against PM proteins may be affected by saponin treatment. For example, on PM of skeletal muscles, labeling efficiency of a glucose transporter (GluT4) was substantially affected by the concentration of saponin [23]. Here, we compared the labeling efficiency of a glutamate receptor (GluR2, an AMPA receptor of subtype 2) on PM between samples permeabilized with saponin and samples treated with 10 min of 50% ethanol followed by several washes of water to clear ethanol off the tissue. For these ethanol-permeabilized samples, saponin was also omitted from all subsequent steps.

**Fig. 3:**
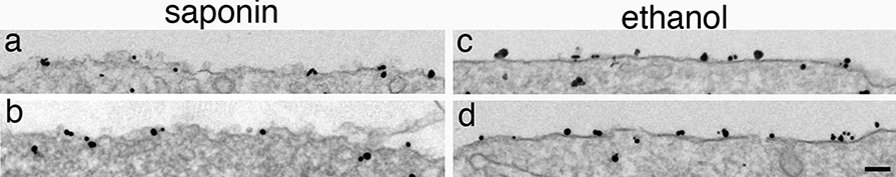
Plasma membranes (PM) of dissociated hippocampal neuronal somas labeled for GluR2, an AMPA type glutamate receptor of the subtype 2. Samples were identically fixed but permeabilized differently with saponin (**a**, **b**) or ethanol (**c**, **d**). Membranes were wavier and labeling density was lower in saponin-treated than in ethanol-treated samples. Scale bar = 100 nm

PM of neuronal soma was specifically labeled with GlurR2 [24] after both permeabilization treatments (Fig. 3). However, PM appeared wavier in saponin-treated samples (Fig. 3a, b) than in ethanol-treated samples (Fig. 3c, d), and the labeling density was lower in saponin-treated samples at 24–55% of those in ethanol-treated samples (Additional file 2).

Based on our experience, antibodies that worked well for immuno-fluorescence LM may not necessarily work for immunogold labeling by EM. The two key factors for EM interpretation are additional fixation and milder permeabilization conditions required to preserve membrane structures. Our recommended initial testing for any antibody is to carry LM experiments with an EM-compatible fixation and permeabilization conditions. For example, samples can be fixed at 4% PF for 15, 30, 60 min initially and then permeabilized with 0.1% saponin for 30 min to see if fluorescence signals survived these conditions. Subsequently, additional modifications on fixation and permeabilization conditions can be tried to balance between achieving better structural preservation and higher labeling efficiency.

It should be noted that Triton X, a popular detergent for immunofluorescence LM, should not be routinely used for EM studies because the biological membranes could be easily dissolved by this detergent and render the EM images uninterpretable. Triton X can only be used under specialized conditions at very low concentration and in conjunction with other additional EM fixatives [25].

### Quality control of secondary antibody

After testing a few different types of secondary antibodies conjugated with gold, our choice reagent is Nanogold-Fab’ from Nanoprobes (Yaphank, NY) due to its higher labeling efficiency [12] and stability over time. This secondary antibody is a Fab’ fragment of the IgG, conjugated with a covalently-bound small gold (1.4 nm) [13]. Although the great majority of Nanogold secondary antibodies we received worked well, a quality check on any new shipment against a known good positive control is still advised. Examples are illustrated in Additional file 3 where sister cultures of hippocampal neurons were identically fixed and permeabilized, labeled with primary antibodies, and subsequently incubated with two different lots of Nanogold secondary antibodies. In two experiments, labeling densities with lot 2 of secondary antibody were only 17–25% of those of lot 1 (Additional file 3). A possible reason for the low efficiency in lot 2 may be low percentage of conjugation of the gold to the secondary antibodies.

We also carried out periodic quality checks on the Nanogold secondary antibodies in our stock, and found that Nanogold antibodies were very stable, even over many years, if stored properly in the refrigerator at 4 °C. Additional file 4 indicates that Nanogold antibodies which had been stored for more than 4 years still yielded similar labeling intensity (80–92%) to recently shipped Nanogold.

Freezing Nanogold is the only handling condition in our hands that caused a substantial decrease in labeling efficiency. Thus, one reason for some lots of Nanogold to perform disappointingly (cf. Additional file 3) could be poor handling *en route*, e. g. accidental freezing during shipment. The typical practice in this lab is to test any new lot of Nanogold secondary antibody with a reliable primary and secondary antibodies as a positive control. Once it passed the test to yield acceptable labeling efficiency, we would buy more of the same lot and keep them for several years. We always have several lots of secondary Nanogold on hand and do not wait until the supplies are very low to buy and test replacements.

### Comparison of labeling efficiency by different silver or gold enhancement reagents

Since the small (1.4 nm) gold particle conjugated to the secondary antibody is not visible by conventional transmission EM, samples need to go through silver or gold enhancement to enlarge the small gold to become visible signals [14, 15]. The efficiency among three different types of silver or gold enhancement reagents are compared here.

The HQ silver enhancement kit from Nanoprobes needs to be handled in a darkroom under a red safety light. We found that it does not require absolute darkness and can be accommodated in a closed room with lights off, and a red safety light on the bench. The silver enhancement kit from Aurion, AURION R-Gent SE-EM, and the gold enhancement kit from Nanoprobes, Goldenhance, are used under regular bench conditions. However, even with the inconvenience of a darkened bench and the safety light requirement, the HQ kit turned out to be our choice reagent due to its higher efficiency and high specificity.

**Fig. 4:**
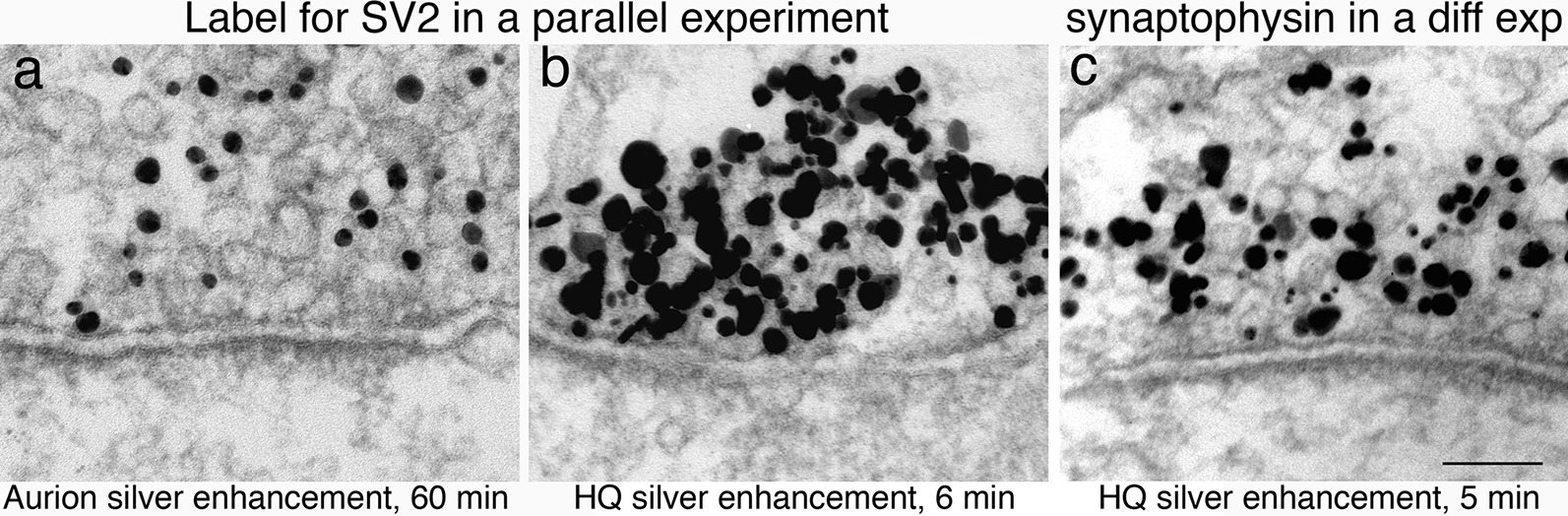
Samples in **a** and **b** were labeled for SV2 and treated identically except for the silver enhancement step. Samples treated with the Aurion silver enhancement kit for 60 min showed signals of more or less uniform-sized particles (**a**), while samples treated with the Nanoprobes HQ silver enhancement kit for 6 min showed signals of heterogeneous sizes at a much higher density (**b**). Sample in **c** was from a separate experiment where sample was labeled with synaptophysin, another SV membrane protein, and the time of HQ silver enhancement was shortened to 5 min, resulting in smaller sized particles. Scale bar = 100 nm

Figure 4a and b illustrate the difference between the HQ kit and the Aurion silver enhancement reagents in a parallel experiment. Hippocampal cultures labeled for SV2, a synaptic vesicle (SV) membrane protein [18], showed specific labeling on SVs in presynaptic terminals with both enhancement reagents. The Aurion enhancement kit yielded homogeneous-sized particles after 60 min of exposure at room temperature (Fig. 4a), the image of which is perfectly suitable for qualitatively illustrating the specific labeling. The Aurion kit also has the convenience that one can monitor the progression of reaction product under a light microscope.

On the other hand, the HQ silver enhancement kit produced heterogeneous-sized particle after 6 min of treatment at room temperature (Fig. 4b). This particular sample in Fig. 4b was clearly over-enhanced that the large and crowded silver particles obscured the underneath structure of the SVs. However, labeling efficiency of the HQ kit was much higher at 266–437% of that of the Aurion kit (Additional file 5). This higher labeling efficiency can be especially advantageous when detecting antigens of low concentration. Notably, the silver enhancement process progresses with time [3] and is temperature-dependent [14]. Thus, the over-sized signals can be remedied by reducing time (Fig. 4c) or temperature of the silver enhancement treatment.

**Fig. 5:**
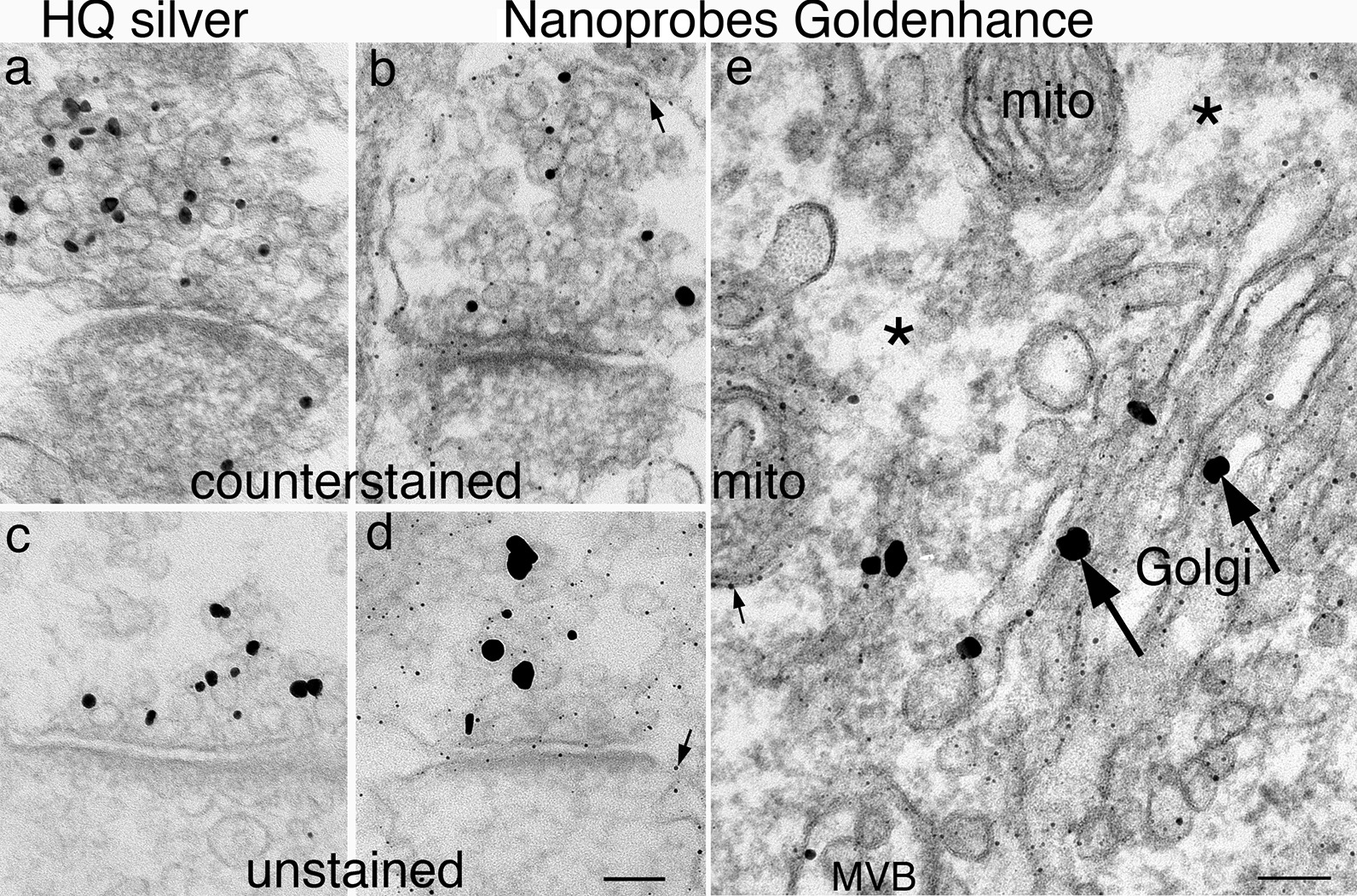
**a**–**d** Synapses from dissociated hippocampal neurons labeled with synapsin I, an SV-associated protein [18]. Thin sections were counterstained in **a** & **b**, and unstained in **c** & **d**. Labeling density on synaptic vesicle clusters is higher after 8 min of HQ silver enhancement (**a**, **c**) than after 10 min of Goldenhance treatment (b, d). Notably, in Goldenhance-treated samples (**b**, **d**), there were fine particles (small arrows) all over membranes in both stained (**b**) and unstained (**d**) sections, indicating that these fine particles are not artifacts of counterstaining. **e** A neuronal soma of perfusion-fixed brain labeled with synaptophysin, known to be present at the Golgi complex [18]. This sample was treated with Goldenhance and only the large particles localized on Golgi (large arrows) are real signals. The fine particles present on membranes of Golgi complex, multivesicular body (MVB), and the inner and outer membranes of mitochondria (small arrow in **e**) are artifacts of the Goldenhance reagents. Asterisks (*) marks the cytosolic area devoid of the fine particles that nonspecifically decorated all membranes. Scale bars = 100 nm. **a**–**d** share the same scale bar

Labeling efficiency and specificity was also tested between the Nanoprobes HQ silver enhancement kit and the Nanoprobes Goldenhance reagents. Samples were identically treated except for the silver (Fig. 5a, c) or gold (Fig. 5b, d) enhancement steps. In two experiments, the labeling efficiency of the HQ kit was higher at 228–416% of that of the Goldenhance kit (Additional file 6).

Regardless of labeling efficiency, the major drawback of the Nanoprobes Goldenhance kit in our hands is that there were nonspecific, fine particles all over membranous structures (Figs. 5b, d, e). These artifacts are distinct from the real signals localized on the SVs (Fig. 5b), and were not caused by counterstaining (Fig. 5d). Figure 5e shows a neuronal soma where membranes of different organelles, including Golgi, mitochondria and multivesicular body, were all decorated with such fine particles. One cautionary sign of the non-specificity of this artifact is that the particles in question are all over every piece of membrane, including the inner and outer membranes of the nuclear envelope and the inner and outer membranes of mitochondria where it is known that the compositions of these membranes are different [26, 27]. Although there were studies that successfully used Goldenhance without such nonspecific artifacts [28], we could not easily get rid of these artifacts and decided to use HQ because of its specificity and higher labeling efficiency.

### Recommendations on managing the HQ silver enhancement kit

Each lot of HQ silver enhancement kit needs to be characterized to determine an optimal development time. Additional file 7a and b show a pair of samples identically treated except for silver enhancement time, resulting in smaller particles with shorter development time. Additional file 7c and d show a pair of parallel samples silver enhanced with two different lots of HQ kits for the same amount of development time, but resulting in different sized particles. Thus, the silver enhancement time should be adjusted for different lots of HQ kits in order to achieve the desired particle sizes.

Our typical practice was to buy several HQ kits of the same lot and store them at – 20 °C. We thawed the HQ reagent, one kit at a time, and refrigerate the unused portion at 4 °C to be used within a month. If the thawed reagents were not expected to be used up within that time frame, we aliquoted the three components of the HQ kit into equal volumes and stored them frozen. The reagents were stable for approximately 1 year in our hands. Longer storage times either in the refrigerator or in the freezer, sometimes changed the optimized development time. We also noticed a trend that the quality of the silver particles tended to have more problems when the HQ reagents used were older.

### Caveats on osmium treatment of silver enhanced samples

**Fig. 6:**
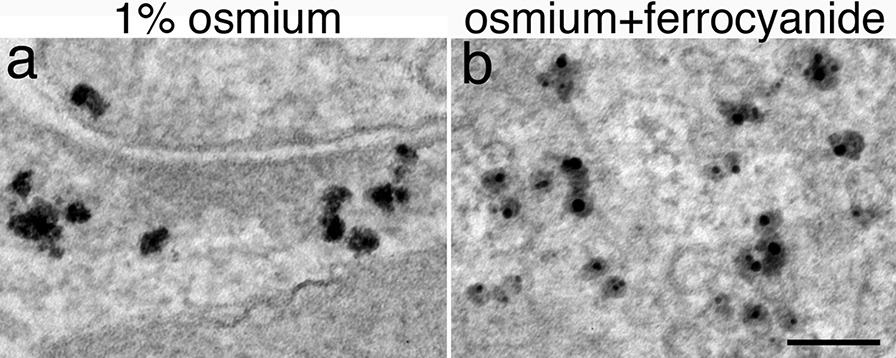
Examples of poor looking silver particles in samples treated with 1 h of 1% osmium tetroxide (**a**) or 1% osmium + 1% potassium ferrocyanide (**b**). Scale bar = 100 nm

Osmium tetroxide treatment is generally required for EM samples in order to preserve membrane structures. However, it can reduce the size of the silver enhanced particles [15]. In some experiments, an osmium tetroxide treatment at 1% for 1 h yielded irregular-shaped particles with a fuzzy border (Fig. 6a). Also, the silver enhanced particles were poorly preserved with “reduced osmium” treatment (Fig. 6b), where potassium ferrocyanide was included with osmium tetroxide.

In order to consistently produce sharp looking silver particles, the routine in our lab now is to use fresh-made 0.2% osmium tetroxide in 0.1 M phosphate buffer at pH 7.4 for 30 min on ice. Because osmium tetroxide is volatile, a 0.2% solution made ahead of time may become less in concentration with time. The consequence of which would be poorly preserved membranes.

One cautionary note is that on rare occasions, the osmium treatment step may bleach the silver enhancement product, resulting in samples with no silver particles at all in the final thin sections. The fact that such samples had absolutely no silver particles anywhere raised concerns because even the control samples where primary antibody was omitted should still have some background particles. Thus, some chemical reactions must have bleached all signals between the steps of silver enhancement and the rest of the processing procedures. We now closely monitor the yellowish/brownish color of silver-enhanced samples by eye while adding the osmium tetroxide solution. We have found that if “bleaching” is to occur, the color would disappear within the first minute. It is not worth proceeding with the “bleached” samples because no signals will be preserved if bleaching occurred at this step. Also, to guard against the possibility that the silver enhancement reagents may contribute to this “bleaching” phenomenon, the repeat experiment would be silver enhanced with a different batch of the HQ kit.

### Caveats on uranyl acetate (UA) en bloc staining

To increase contrast, tissues are often *en bloc* stained with UA following osmium treatment and prior to dehydration. This UA *en bloc* staining was carried out at cold temperature (4 °C) under light-tight conditions either wrapped in foil or kept in the refrigerator. Although this step is not necessary, we found the increased contrast in immunogold-labeled samples, which were typically weakly fixed with lesser structural preservation, worthy of this additional step.

**Fig. 7:**
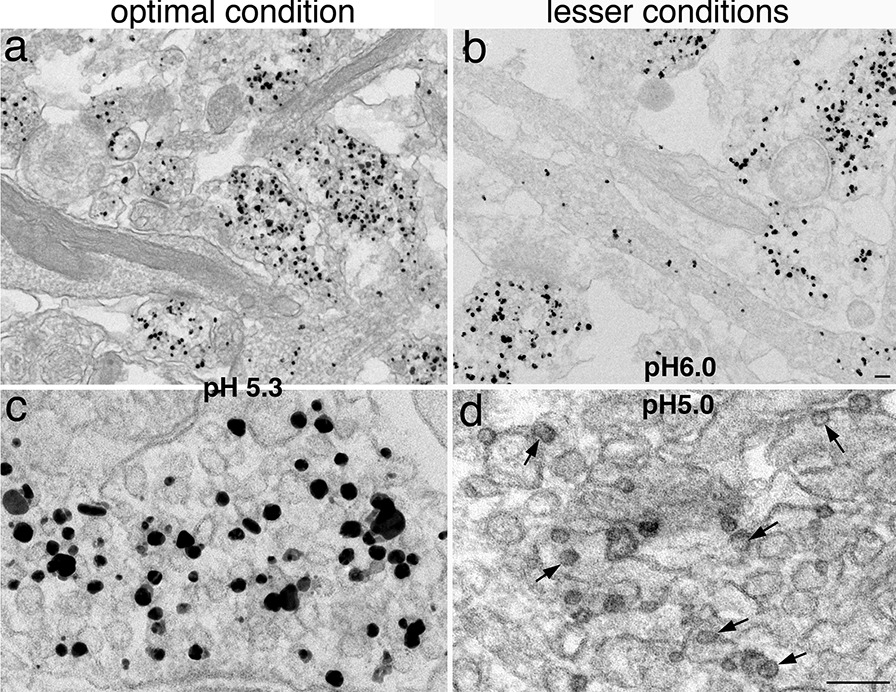
Dissociated hippocampal cultures labeled with SV2. Images had more contrast when samples were en bloc stained with UA at pH 5.3 (**a**) than at pH 6.0 (**b**). Contrast of the image was even stronger when the sample was treated with UA at pH 5.0 (**d**), but the silver particles became grey shadows (small arrows in **d**) when compared to the black particles in samples treated with UA at pH 5.3 (**c**). Scale bars = 100 nm. **a** & **b** shared the same bar, **c** & **d** shared the same bar

Our routine was to make a stock 0.2 N acetate buffer at pH 5.0, then make the final solution of 1% UA in 0.1 N buffer, the pH of which became 5.3. An attempt to readjust the pH to 5.0 of the UA solution resulted in damaging the appearance of silver particles (small arrows in Fig. 7d). The particles became light grey, and appear to be on the verge of vanishing. Thus, the UA *en bloc* staining may also contribute to the disappearing of the silver particles. Adjusting the pH to 6.0 protects the integrity of the silver particles (Fig. 7b), but the contrast of the sample became so low that there is no longer any benefit of this UA *en bloc* staining step.

Because “greyish” silver particles were still occasionally seen in samples treated with 1% UA in 0.1 N acetate buffer at pH 5.3, we tried lowering the concentration and treatment time of the UA solution. In one parallel experiment, samples labeled with SynGAP [29] and treated with 0.25% UA for overnight had a higher contrast and better preservation of the membranes (Additional file 8a) than samples treated for 1 h (Additional file 8b). However, the overnight-treated samples appeared to have a lower labeling efficiency. We suspected that some of the silver-enhanced particles disappeared during the overnight treatment. Thus, our routine treatment now is to *en bloc* stain with 0.25% UA for 1 h at 4 °C.

### Quality of silver particles related to sectioning and counter staining

**Fig. 8:**
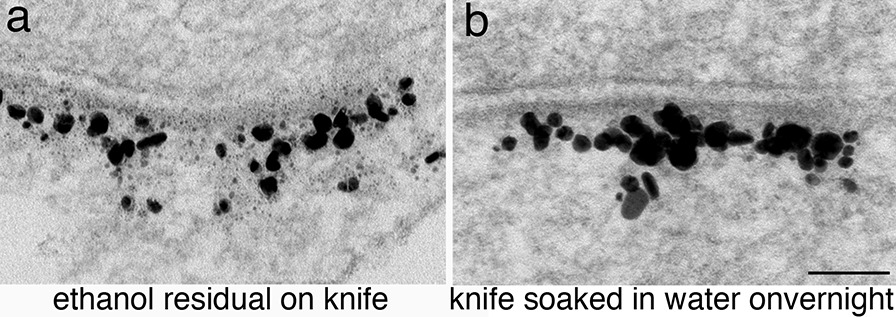
Dissociated hippocampal cultures labeled for Homer 1b/c, a scaffold protein at the PSD [30]. When sections were cut with a diamond knife cleaned with ethanol, the silver particles had a smudged appearance, with numerous fine dots in the vicinity of the particles (**a**). In contrast, the silver particles appeared intact (**b**) in sections from the same block that was cut with a diamond knife never cleaned with ethanol. Scale bar = 100 nm

Over the years, we noticed that the silver particles in thin sections of some samples appeared smudged as if the silver diffused out of the particles (Fig. 8a). Eventually, we traced this smudging of silver particles to the cleaning of the diamond knife used to cut thin sections. For example, the same block cut with two different diamond knives resulted in different looking silver particles. A knife that had been cleaned with ethanol prior to cutting thin sections produced smudged silver particles (Fig. 8a), while another knife that was never cleaned with ethanol produced sharp particles (Fig. 8b). We eventually confirmed that this ethanol-induced smudging of silver particles can be remedied by soaking the ethanol-cleaned knife in water over night.

Notably, a detergent-cleaned diamond knife produced similar looking smudged silver particles, and the smudging effect left by the liquid soap is extremely difficult to remove even after repeated rinsing and prolonged soaking in water. Additionally, despite our careful management on the diamond knives, we sometimes still found smudged silver particles, especially in brain tissues. We suspect that the baskets used to hold these tissues during silver enhancement procedures might have been cleaned with detergent, thus, smudged the silver particles.

By reexamining archived grids from past years, it is clear that the quality of the silver-enhanced particles in thin sections all eventually deteriorated with time. A few examples are shown in Additional file 9. The shape of the silver enhanced particle was typically roundish with sharp edges immediately after sectioning (Additional file 9, left column), but deteriorated into irregular shaped, fuzzy particles with time (Additional file 9, right column). The degree of deterioration typically progressed with time, but both the speed and degree of deterioration were variable among different experiments, probably due to the different batches and/or age and handling of silver enhancement kits. Counterstaining of thin sections appeared to somewhat protect the silver particles from deteriorating and could prolong the usable life of the thin sectioned grids.

In order to image the silver particles at their best condition, we counterstained thin sections the next day after they were cut, and examined the stained sections as soon as possible, at least within days. However, if the silver particles deteriorated in the sections, the same block can be cut further for more thin sections. The deterioration of the silver particles appeared to be related to the exposed cut face of the block because the initial thin sections recut from the face of a previously cut block also contained deteriorated silver particles, yet the deeper sections of the same block yielded intact looking particles.

### Considerations on interpretation of immunogold labeling results

One of the crucial issues for any immunolabeling study is the specificity of the antibody used to label the protein of interest. Many different validation protocols are summarized in reviews [31, 32], but cross-reactive antibodies still pose problems due to lack of multiple approaches in verifications.

**Fig. 9:**
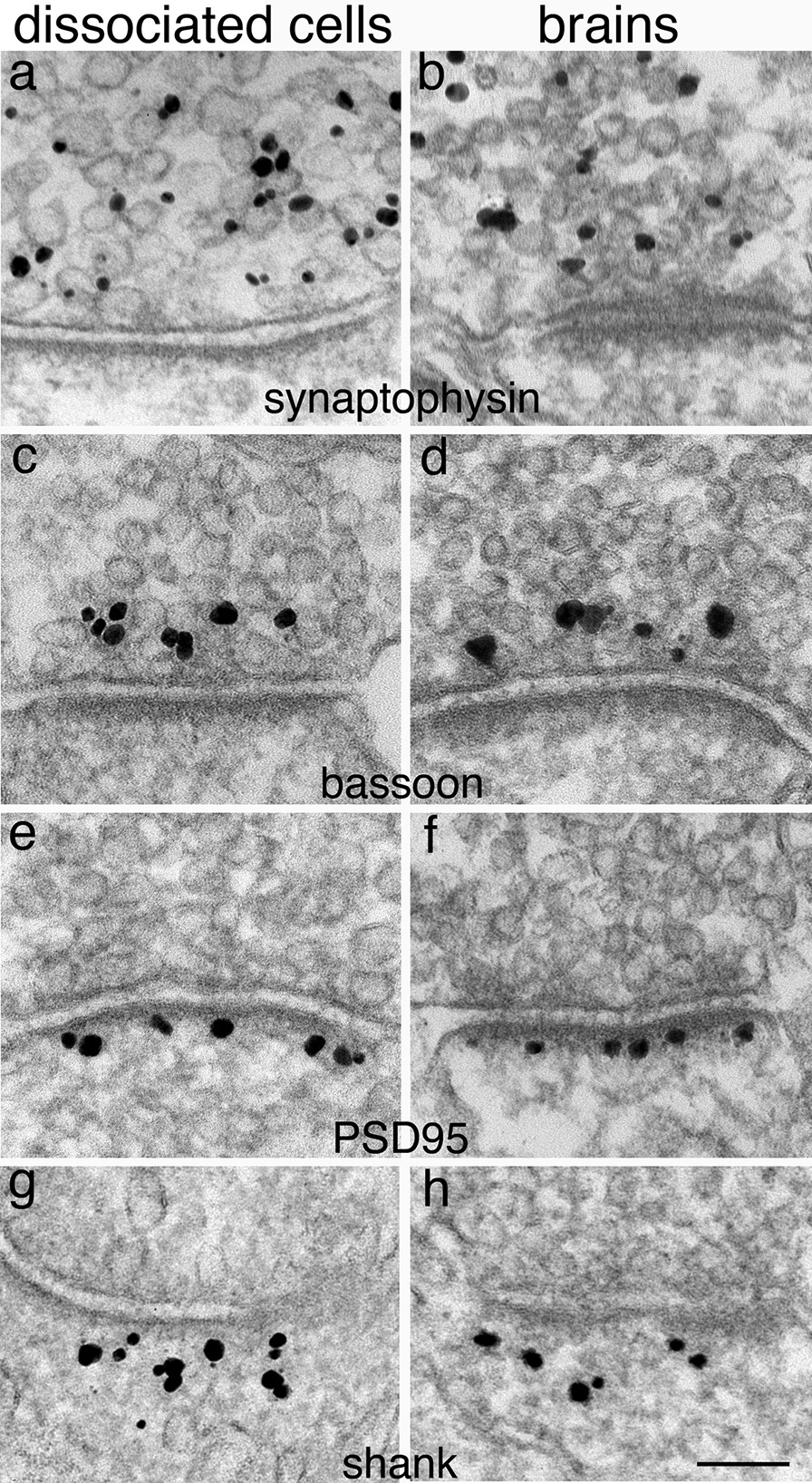
Dissociated hippocampal culture (left column) and perfusion-fixed mouse or rat brains (right column) labeled with different antibodies. Synaptophsin is localized to synaptic vesicles (**a**, **b**), bassoon is localized to active zone cytomatrix (**c**, **d**), PSD95 is localized within 30 nm of the postsynaptic membrane (**e**, **f**), and shank is localized within 40–120 nm of the postsynaptic membrane (**g**, **h**). Scale bar = 100 nm

For immunogold labeling studies at the EM level, the simplest way to verify the specificity of an antibody is to carry a control sample where the primary antibody is omitted. In these control samples, there should only be background particles scattered randomly. If a primary antibody worked, specific signals should be localized to the expected sites with high precision. Furthermore, immunogold labeling at the EM level has an advantage of being able to use different antibodies to serve as controls for each other. For example, four different antibodies in Fig. 9 were each specifically localized to different sites which may be difficult to differentiate at the LM level, but can be unequivocally distinguished at the EM resolution level. Synaptophysin, an SV membrane protein, was localized to SVs in presynaptic terminals (Fig. 9a, b) [18], while bassoon, an active zone cytomatrix protein, is localized at the active zone within 100 nm of the presynaptic membrane (Fig. 9c, d) [17]. On the postsynaptic side, PSD95, a postsynaptic density protein, is localized immediate adjacent to the postsynaptic membrane (Fig. 9e, f) [5], while Shank, a PSD scaffold protein, is located to the deeper layer of the PSD (Fig. 9g, h) [21]. Each of these antibodies was specifically localized to their expected sites without cross-labeling other cellular elements.

**Fig. 10:**
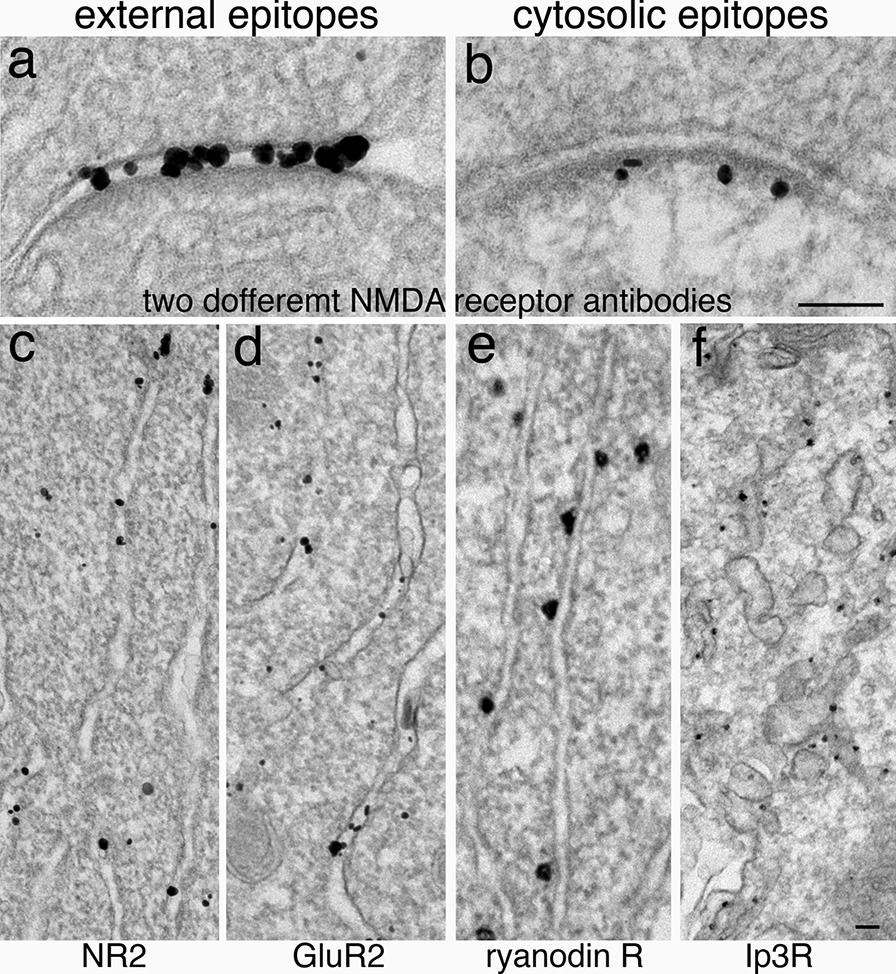
Verification of specificity of antibodies made against different epitopes of transmembrane proteins. Antibodies made against extracellular (external) epitopes should be localized to the extracellular side of the postsynaptic membrane (**a**) and the lumen of the ER (**c**, **d**), while antibodies made against cytosolic epitopes should be localized to the cytosolic side of the postsynaptic membrane (**b**) and the cytosolic side of the ER (**e**, **f**). **a**–**e** are from dissociated hippocampal cultures, and **f** is from Purkinje soma of perfusion-fixed cerebellum. Scale bars = 100 nm, **a** & **b** share the same bar, **c**-**f** share the same bar

Another advantage of immunogold labeling at the EM level is the precise localization of transmembrane proteins to their epitopes on either side of the membrane. For example, one antibody made against the N-terminus of the NMDA receptor (NR2B subunit) was localized to the extracellular side of the postsynaptic membrane (Fig. 10a) [33], while another antibody made against the C-terminus was localized to the cytosolic side of the membrane (Fig. 10b). The same extracellular NR2B antibody was correctly localized to the lumen of the endoplasmic reticulum (ER, Fig. 10c), so was another antibody against the N-terminus of an AMPA receptor, GluR2 (Fig. 10d) [24]. Two other antibodies made against the cytosolic epitopes of ER membrane proteins, ryanodine receptor and Ip3 receptor, were localized to the cytosolic side of the ER membranes (Fig. 10e, f) [34].

**Fig. 11:**
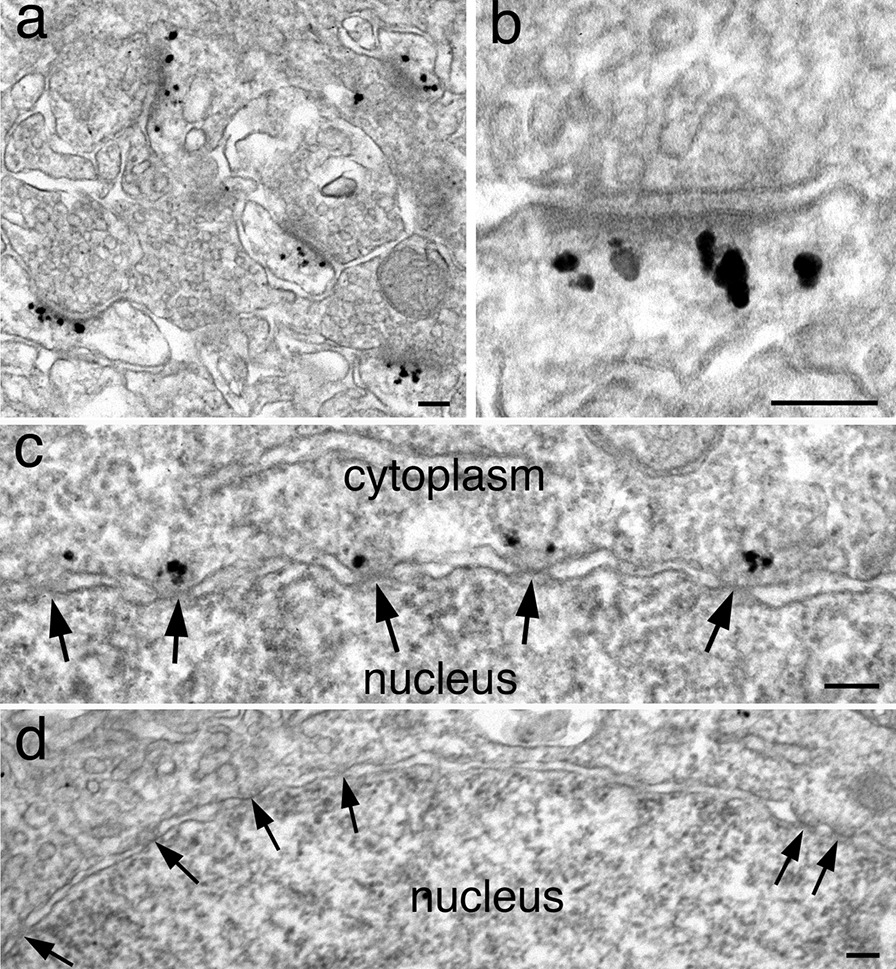
Perfusion-fixed mouse brains labeled with Homer 2 (**a**–**c**) and Homer 1/2/3 (**d**). Label for a Homer 2 antibody was specifically localized at the PSDs with very little background elsewhere (**a**), and correctly situated at the deeper layer of the PSD (**b**). Additional signals of this antibody were also seen associated with the cytoplasmic side of the nuclear pores (arrows in **c**). However, another pan Homer antibody was not detected at the nuclear pore (**d**). Scale bars = 100 nm

It should be cautioned that some antibodies could cross react to additional, unintended epitopes [32]. For example, one antibody against the Homer 2 subfamily of the homer protein, a PSD scaffold protein [30], was correctly localized to the deeper layer of the PSD (Fig. 11a, b). However, this antibody was also localized to other locations unrelated to the expected distribution of Homer proteins (Fig. 11c). Signals were seen on the cytosolic side of the nuclear envelope, associated with the nuclear pores (arrows in Fig. 11c). It was determined that this nuclear pore labeling is a cross reaction unrelated to the Homer 2 protein because another pan Homer antibody which labels all 3 subfamilies of the Homer protein did not label the nuclear pore (Fig. 11d). Nevertheless, this Homer 2 antibody is still useful at the EM level because its labeling of the PSD is specific and undisturbed by the additional, non-specific labeling of the nuclear pores, which is distant and distinct from the PSDs.

**Fig. 12:**
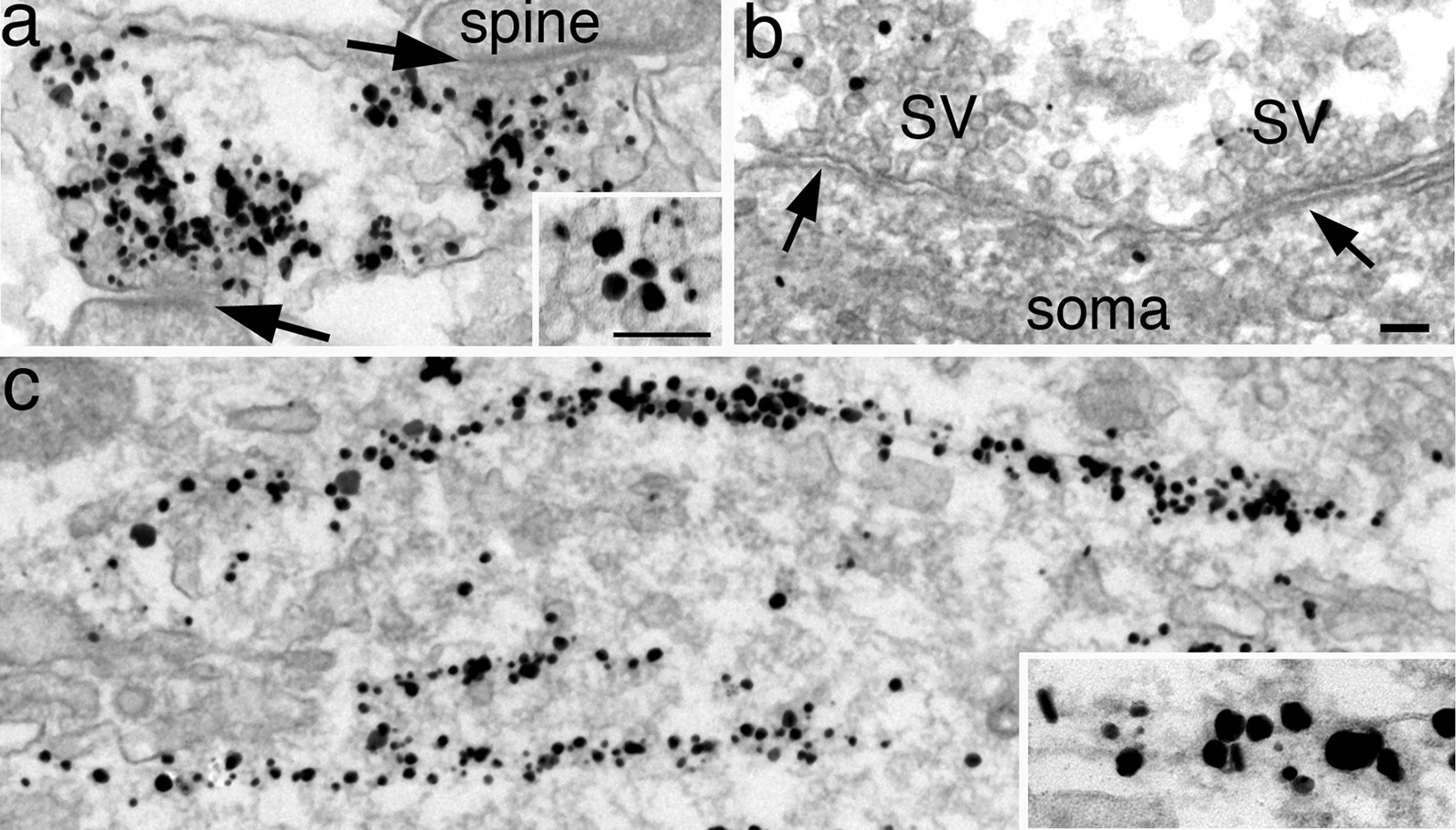
Dissociated hippocampal cultures labeled with a VGluT ab. Label was correctly localized to SVs of glutamatergic terminals (**a**, arrows point to the characteristic PSDs of asymmetric synapses), but not to SVs of GABAergic terminals of symmetric synapses (arrows in **b**). However, this antibody also cross reacted with a filamentous structure in the cytoplasm (**c**). Scale bars = 100 nm. **a**–**c** share the same bar; insets of **a** & **c** share the same bar

Another example of an antibody localized to unintended organelles is shown in Fig. 12. An antibody against VGluT (vesicular glutamate transporter), an SV membrane protein specific for glutamate uptake [35], was correctly localized to SVs of glutamatergic synaptic terminals (Fig. 12a) and not to SVs of GABAergic inhibitory terminals (Fig. 12b). However, signals were also detected on cytosolic filaments in soma/dendrites (Fig. 12c). Since VGluT is an integral membrane protein, the label on filaments (inset of Fig. 12c) must be a cross reaction of this antibody and cannot represent VGluT localization. Although the localization on SV membranes was correct (inset of Fig. 12a), we decided not to use this antibody because the cross reaction was too widespread and can potentially obscure the correct signals.

### Quantification considerations

**Fig. 13:**
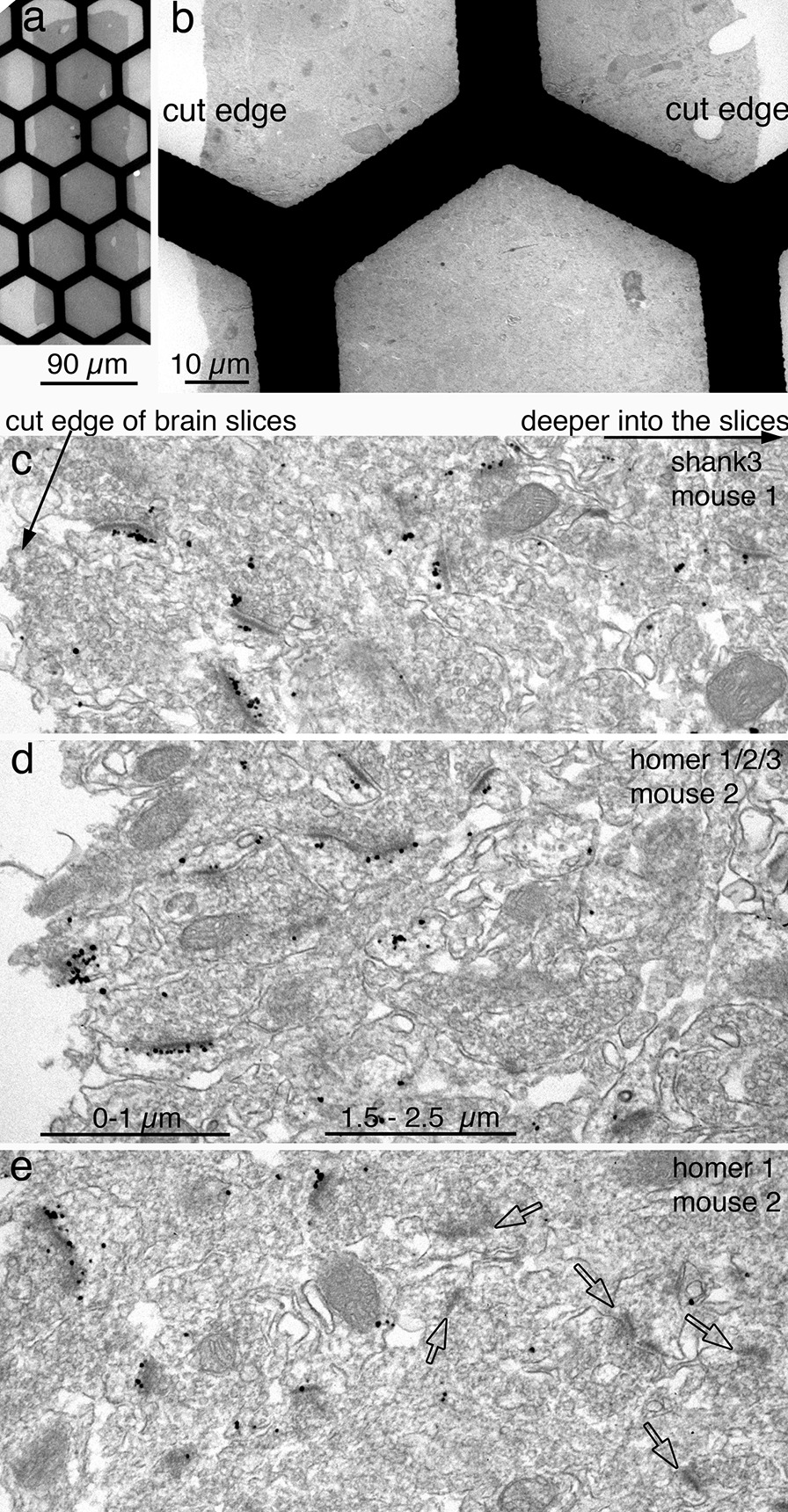
A cross-sectioned 90 µm thick brain slice is collected on a 400-mesh hexagonal grid (**a**), and the two cut edges of the slice are enlarged in (**b**). Perfusion-fixed brain slices labeled with shank 3 (**c**), homer 1/2/3 (**d**), and homer 1 (**e**), three different antibodies against these PSD scaffold proteins [21, 30]. Labeling density decreases from the cut edges of the slices on left toward the deeper tissue of the slices on right (**c**–**e**). The penetration gradient is similar between **c** and **d** where two antibodies were used on slices from two different mice. However, depth of penetration for the third antibody (**e**) appeared to be shallower, even though the slice was from the same animal as in (**d**). Many PSDs were unlabeled (open arrows in **e**) beyond 2 µm deep from the cut edge. The two bars on bottom of d were each 1 µm long and marked the depth from the cut edge of the slice

One of the advantages of immunogold labeling is that the labeling density is quantifiable by counting individual particles [8–10, 12]. However, several issues can affect the validity of quantification. For example, immunolabeling reagents can typically penetrate throughout the monolayer cells of dissociated cultures, but brain tissues present a penetration issue due to tightly packed processes and narrow extracellular space, both of which are not conducive to penetration and diffusion of reagents. To guard against this penetration gradient issue, our routine practice is to cut cross sections of immunogold-labeled brain slices, and to only sample from the cut edges for labeling density (Fig. 13).

Labeling gradient from the cut edges toward the center of the slices cannot be easily assessed from semi-thin sections by LM, but can only be accurately assessed by EM. Although the bars of the meshed grids impede examination of the entire cut edge of the brain slices (Fig. 13a, b), 400-meshed grids are still preferred over single slot grids because meshed grids already offers enough exposed areas for necessary sampling. Furthermore, single slot grids require additional labor of film-coating, which in itself may introduce artifacts if not done carefully. Such a labor-intensive effort involving single slot grids is only reserved for projects that requires serial sections [18].

There is a steep downward gradient of label from the cut edge of the tissue toward the center of the brain slices (Fig. 13c–e). Although the depth of labeling and the steepness of the gradient can vary by different perfusion fixations or by different primary antibodies, the highest labeling density was consistently seen in the most superficial 1–2 µm of the brain slices. Thus, for our quantification projects using perfusion-fixed brains, sampling is restricted to within 1 µm of the cut edges. It would not be a valid comparison if sampling were collected from different depth of the slices where labeling efficiencies are vastly different.

**Fig. 14:**
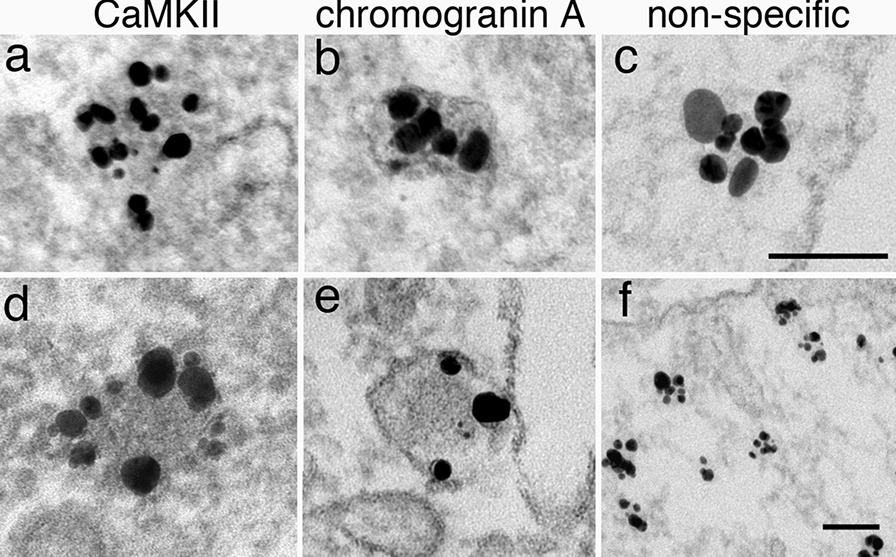
Dissociated hippocampal cultures labeled with CaMKII (**a**, **d**), chromogranin A (**b**, **e**) and two non-specific antibodies (**c**, **f**). The clusters of signals in the left two columns showed legitimate multiple signals of the aggregated proteins. The two primary antibodies used in (**c**, **f**) turned out to be non-specific, but these images illustrated clumping of the secondary antibody, and therefore, these clusters should not be counted as multiple signals. Scale bars = 100 nm

Another quantification issue is in the interpretation of aggregated signals. Tightly aggregated proteins can appear as dark material legitimately labeled with multiple signals. For example, CaMKII is shown to self-aggregate into clusters (Fig. 14a, d) [36], and chromogranin A is known to aggregate in high concentrations in the dense core of dense core vesicles (Fig. 14b, e) [37]. However, tightly clustered particles could be artifacts, especially when there was no evidence of protein aggregates association with these clusters of particles (Fig. 14c, f). Such clustering of particles is likely caused by clumping of secondary antibodies and could be miscounted as multiple signals. This possibility can be easily tested by using a known, good secondary antibody in parallel experiments for comparison. The lot of secondary antibody with high degree of clumping should be discarded because it cannot be used for quantification of labeling density.

Interestingly, the protein aggregates in some samples appeared denser than others, and the core of the aggregates lacked labeling (Fig. 14d, e). The CaMKII cluster in (Fig. 14d) was from a sample treated with NMDA for 3 min, where CaMKII molecules might have formed a very tight aggregation that impeded the penetration of labeling reagents. Whereas the CaMKII cluster in (Fig. 14a) was from a sample treated with glycine for 2 min, where the CaMKII molecules could be aggregated with a looser configuration, more conducive to penetration of reagents. On the other hand, the dense core in (Fig. 14e) was from a 6-day old culture, where the dense core vesicles could be more mature and the dense core was more densely packed than the ones in (Fig. 14b), which were from a 3-day old culture. Nevertheless, these examples illustrate that counting labels from Fig. 14d and e may not truly reflect the number of epitopes present in these tightly packed protein aggregates.

## Conclusions

In conclusion, the present paper presented means to optimize the fixation and other treatment conditions for a pre-embedding immunogold labeling technique, summarized potential pitfalls and remedies of this technique, and discussed caveats on interpretation of labeling results. If carried out carefully, this method is still one of the best tools in localizing specific proteins at the ultrastructural level, and in studying redistribution of proteins in neuronal tissues under different stimulation conditions.

## Supplementary Information

**Additional file 1.** Fixation time affects labeling efficiency.

**Additional file 2.** Labeling density (mean ± SEM) of label for GluR2 on plasma membrane of neuronal soma permeabilized with saponin or ethanol.

**Additional file 3.** Quality of the secondary antibody affects the labeling efficiency.

**Additional file 4.** Labeling density (mean ± SEM) of IRSp53 (exp 1 & 2) or SV2 (exp 3) in hippocampal cultures incubated with different lots of secondary antibodies of different storage time.

**Additional file 5.** Labeling density (mean ± SEM) of SV2 (exp 1 & 2) or synaptophysin (exp 3) after Nanoprobes HQ silver enhancement or Aurion silver enhancement kit.

**Additional file 6.** Labeling density (mean ± SEM) of two synaptic vesicle proteins treated with Nanoprobes HQ silver enhancement or Nanoprobes Goldenhance kit.

**Additional file 7.** The size of silver-enhanced particles depends on development time and varies among different lots of reagents.

**Additional file 8.** Uranyl acetate *en bloc* treatment time affects labeling efficiency.

**Additional file 9.** Quality of silver particles in thin sections deteriorates over time.

## Data Availability

The datasets generated and/or analyzed during the current study are available from the corresponding author on reasonable request.
