# Supplementary material for: Optimization of protocols for pre-embedding immunogold electron microscopy of neurons in cell cultures and brains
Source: Mol Brain. 2021 Jun 3;14:86. doi: 10.1186/s13041-021-00799-2 (PMC8173732; doi:10.1186/s13041-021-00799-2)
Supplement: Supplementary file 1 — Additional file 1. Fixation time affects labeling efficiency. [file 13041_2021_799_MOESM1_ESM.docx]

**Additional File 1. Fixation time affects labeling efficiency.**


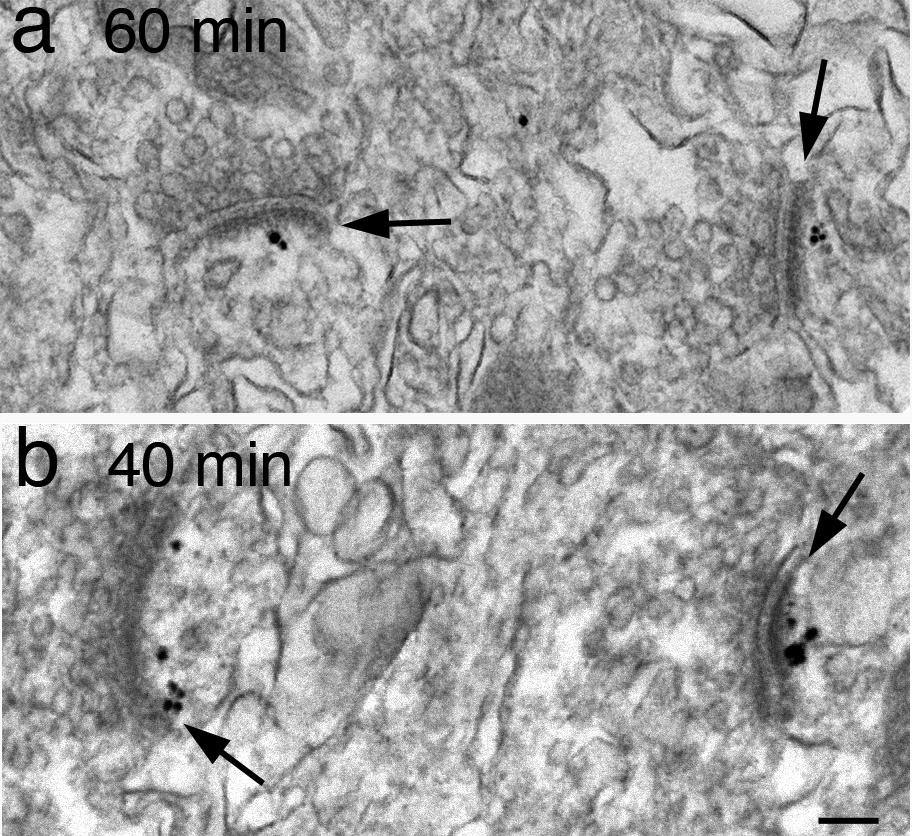


Perfusion-fixed mouse brains labeled for Shank 3, a postsynaptic density (PSD) scaffold protein [21]. Labeling density at PSDs (arrows) was lower in the brain that was fixed with 4% PF for 60 min (a) than the one that was fixed for 40 min (b). Scale bar = 100 nm.
