# Supplementary material for: Optimization of protocols for pre-embedding immunogold electron microscopy of neurons in cell cultures and brains
Source: Mol Brain. 2021 Jun 3;14:86. doi: 10.1186/s13041-021-00799-2 (PMC8173732; doi:10.1186/s13041-021-00799-2)
Supplement: Supplementary file 2 — Additional file 2. Labeling density (mean ± SEM) of label for GluR2 on plasma membrane of neuronal soma permeabilized with saponin or ethanol. [file 13041_2021_799_MOESM2_ESM.docx]

**Additional File 2. Labeling density (mean ± SEM) of label for GluR2 on plasma membrane of neuronal soma permeabilized with saponin or ethanol.**

|  | **Saponin** | **Ethanol** | **%**  **saponin / ethanol** |
| --- | --- | --- | --- |
| **Exp 1** | 1.1 ± 0.1 (45) | 1.8 ± 0.1 (66)  P<0.0001 | 40% |
| **Exp 2** | 1.0 ± 0.3 (11) | 2.2 ± 0.3 (21)  P<001 | 55% |
| **Exp 3** | 0.32 ± 0.06 (22) | 1.36 ± 0.16 (25)  P<0.0001 | 24% |

• GluR2 is a glutamate receptor of the AMPA subtype 2 [24]. Labeling density = number of labels per µm plasma membrane of neuronal soma.

• (n) = number of neuronal soma sampled.

• Values within each experiment was tested by Student’s t-test.
