# Supplementary material for: Optimization of protocols for pre-embedding immunogold electron microscopy of neurons in cell cultures and brains
Source: Mol Brain. 2021 Jun 3;14:86. doi: 10.1186/s13041-021-00799-2 (PMC8173732; doi:10.1186/s13041-021-00799-2)
Supplement: Supplementary file 3 — Additional file 3. Quality of the secondary antibody affects the labeling efficiency. [file 13041_2021_799_MOESM3_ESM.docx]

**Additional File 3. Quality of the secondary antibody affects the labeling efficiency.**


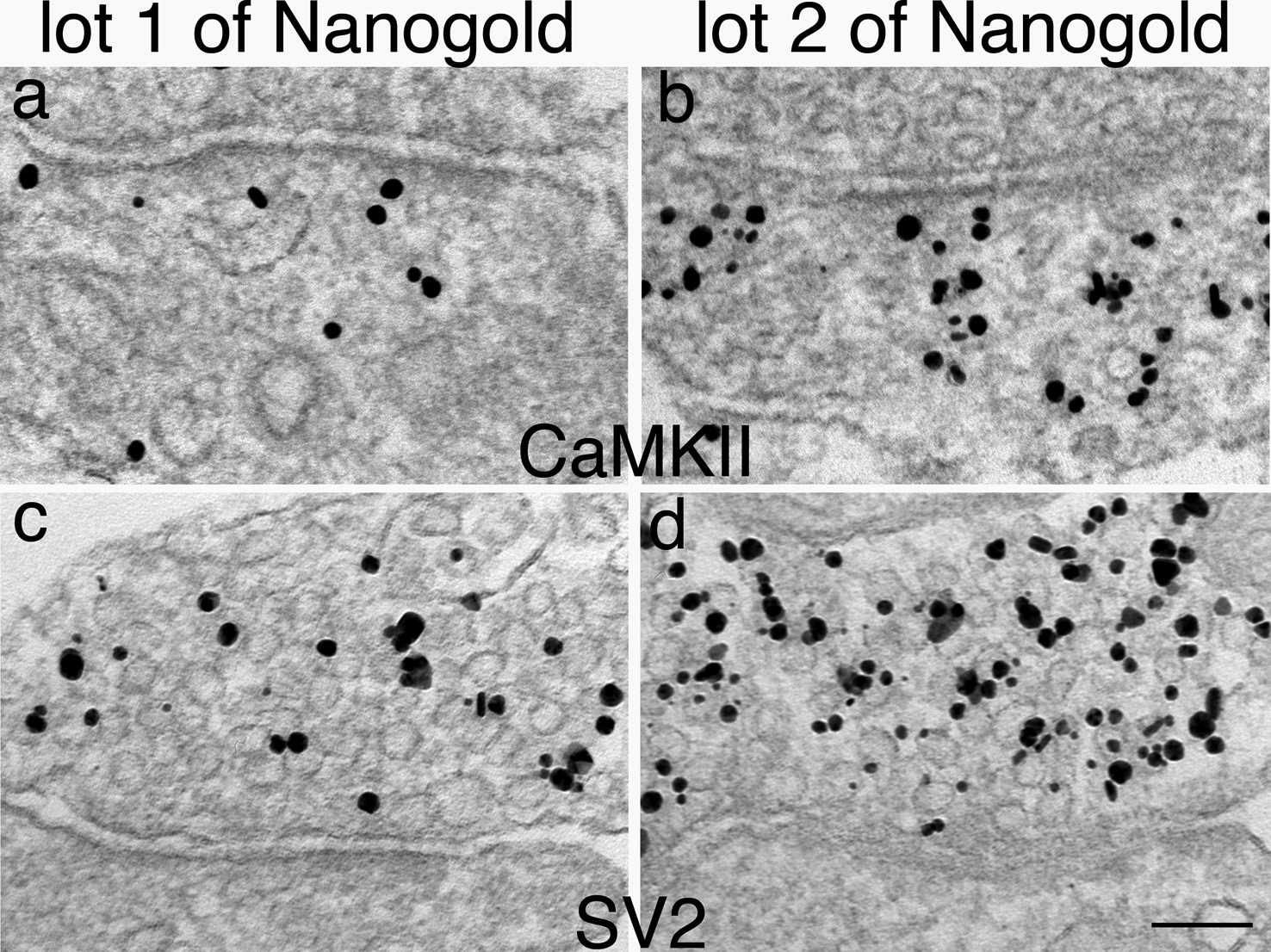


Dissociated hippocampal cultures were labeled for CaMKII (a, b), or SV2 (d, e), then incubated with two different lots of secondary antibodies. Scale bars = 100 nm.

**Labeling densities (mean ± SEM) were significantly lower with lot 2 than with lot 1.**

|  | **Lot 1** | **Lot 2** | **%**  **lot 2 / lot 1** |
| --- | --- | --- | --- |
| **Exp 1**  **CaMKII** | 47.7 ± 5.4 (22) | 8.1 ± 1.6 (15) | 17%  P<0.0001 |
| **Exp 2**  **SV2** | 550 ± 22 (39) | 138 ± 8 (39) | 25%  P<0.0001 |

• CaMKII is a cytosolic protein also present at the PSD [6]. Labeling density = number of labels within 120 nm of the postsynaptic membrane per µm PSD length.

• SV2 is a synaptic vesicle (SV) membrane protein [18]. Labeling density = number of labels per µm^2^ of SV cluster area in presynaptic terminals.

• (n) = number of synaptic profiles measured.

• Values within experiment tested by Student t-test.
