# Supplementary material for: Optimization of protocols for pre-embedding immunogold electron microscopy of neurons in cell cultures and brains
Source: Mol Brain. 2021 Jun 3;14:86. doi: 10.1186/s13041-021-00799-2 (PMC8173732; doi:10.1186/s13041-021-00799-2)
Supplement: Supplementary file 4 — Additional file 4. Labeling density (mean ± SEM) of IRSp53 (exp 1 & 2) or SV2 (exp 3) in hippocampal cultures incubated with different lots of secondary antibodies of different storage time. [file 13041_2021_799_MOESM4_ESM.docx]

**Additional File 4. Labeling density (mean ± SEM) of IRSp53 (exp 1 & 2) or SV2 (exp 3) in hippocampal cultures incubated with different lots of secondary antibodies of different storage time.**

|  | **lot 1**  **(fresh shipment)** | **lot 2**  **(stored > 4 years )** | **%**  **lot 2 / lot 1** |
| --- | --- | --- | --- |
| **Exp 1, IRSp53 ab1**  Goat-anti-Mouse Fab’ | 36.5 ± 4.3 (41) | 29.2 ± 2.0 (22) | 80%  N. S. |
| **Exp 2, IRSp53 ab2**  Goat-anti-rabbit Fab’ | 27.2 ± 2.4 (37) | 25.0 ± 1.7 (13) | 92%  N. S. |
| **Exp 3, SV2**  Goat-anti-Mouse Fab’ | 550 ± 22 (39) | 451 ± 17 (43) | 82%  P<0.001 |

• IRSp53 is an actin-associated protein enriched at the PSD [20]. Labeling density = number of labels within 120 nm of the postsynaptic membrane per µm PSD length.

• SV2 is a synaptic vesicle (SV) membrane protein [18]. Labeling density = number of labels per µm^2^ of SV cluster area in presynaptic terminals.

• (n) = number of PSD or presynaptic terminal profiles measured.

• Values within experiment tested by Student t-test. N. S., not significant.
