# Supplementary material for: Optimization of protocols for pre-embedding immunogold electron microscopy of neurons in cell cultures and brains
Source: Mol Brain. 2021 Jun 3;14:86. doi: 10.1186/s13041-021-00799-2 (PMC8173732; doi:10.1186/s13041-021-00799-2)
Supplement: Supplementary file 5 — Additional file 5. Labeling density (mean ± SEM) of SV2 (exp 1 & 2) or synaptophysin (exp 3) after Nanoprobes HQ silver enhancement or Aurion silver enhancement kit. [file 13041_2021_799_MOESM5_ESM.docx]

**Additional File 5. Labeling density (mean ± SEM) of SV2 (exp 1 & 2) or synaptophysin (exp 3) after Nanoprobes HQ silver enhancement or Aurion silver enhancement kit.**

|  | **HQ** | **Aurion** | **%**  **HQ / Aurion** |
| --- | --- | --- | --- |
| **Exp 1**  **SV2** | 789 ± 117 (7) | 297 ± 39 (11) | 266%  P<0.005 |
| **Exp 2**  **SV2** | 942 ± 37 (10) | 249 ± 21 (8) | 378%  P<0.0001 |
| **Exp 3**  **synaptophysin** | 1031 ± 71 (15) | 236 ± 19 (20) | 437%  P<0.0001 |

• SV2 and synaptophysin are two different synaptic vesicle (SV) membrane proteins [18]. Labeling density = number of particles per µm^2^ of SV cluster area in presynaptic terminals.

• (n) = number of presynaptic terminals measured.

• Values within experiment tested by Student t-test.
