# Supplementary material for: Optimization of protocols for pre-embedding immunogold electron microscopy of neurons in cell cultures and brains
Source: Mol Brain. 2021 Jun 3;14:86. doi: 10.1186/s13041-021-00799-2 (PMC8173732; doi:10.1186/s13041-021-00799-2)
Supplement: Supplementary file 6 — Additional file 6. Labeling density (mean ± SEM) of two synaptic vesicle proteins treated with Nanoprobes HQ silver enhancement or Nanoprobes Goldenhance kit. [file 13041_2021_799_MOESM6_ESM.docx]

**Additional File 6. Labeling density (mean ± SEM) of two synaptic vesicle proteins treated with Nanoprobes HQ silver enhancement or Nanoprobes Goldenhance kit.**

|  | **HQ** | **Goldenhance** | **%**  **HQ / Goldenhance** |
| --- | --- | --- | --- |
| **Exp 1**  **Synapsin I** | 158 ±12 (19) | 38 ± 7 (10) | 416%  P<0.0001 |
| **Exp 2**  **SV2** | 249 ± 13 (27) | 109 ± 16 (13) | 228%  P<0.0001 |

• Synapsin I is a synaptic vesicle (SV)-associated protein, and SV2 is an SV membrane protein [18].

• Labeling density = number of particles per µm^2^ of SV cluster area in presynaptic terminals.

• (n) = number of presynaptic terminals measured.

• Values within experiment tested by Student T-test.
