# Supplementary material for: Optimization of protocols for pre-embedding immunogold electron microscopy of neurons in cell cultures and brains
Source: Mol Brain. 2021 Jun 3;14:86. doi: 10.1186/s13041-021-00799-2 (PMC8173732; doi:10.1186/s13041-021-00799-2)
Supplement: Supplementary file 7 — Additional file 7. The size of silver-enhanced particles depends on development time and varies among different lots of reagents. [file 13041_2021_799_MOESM7_ESM.docx]

**Additional File 7. The size of silver-enhanced particles depends on development time and varies among different lots of reagents.**


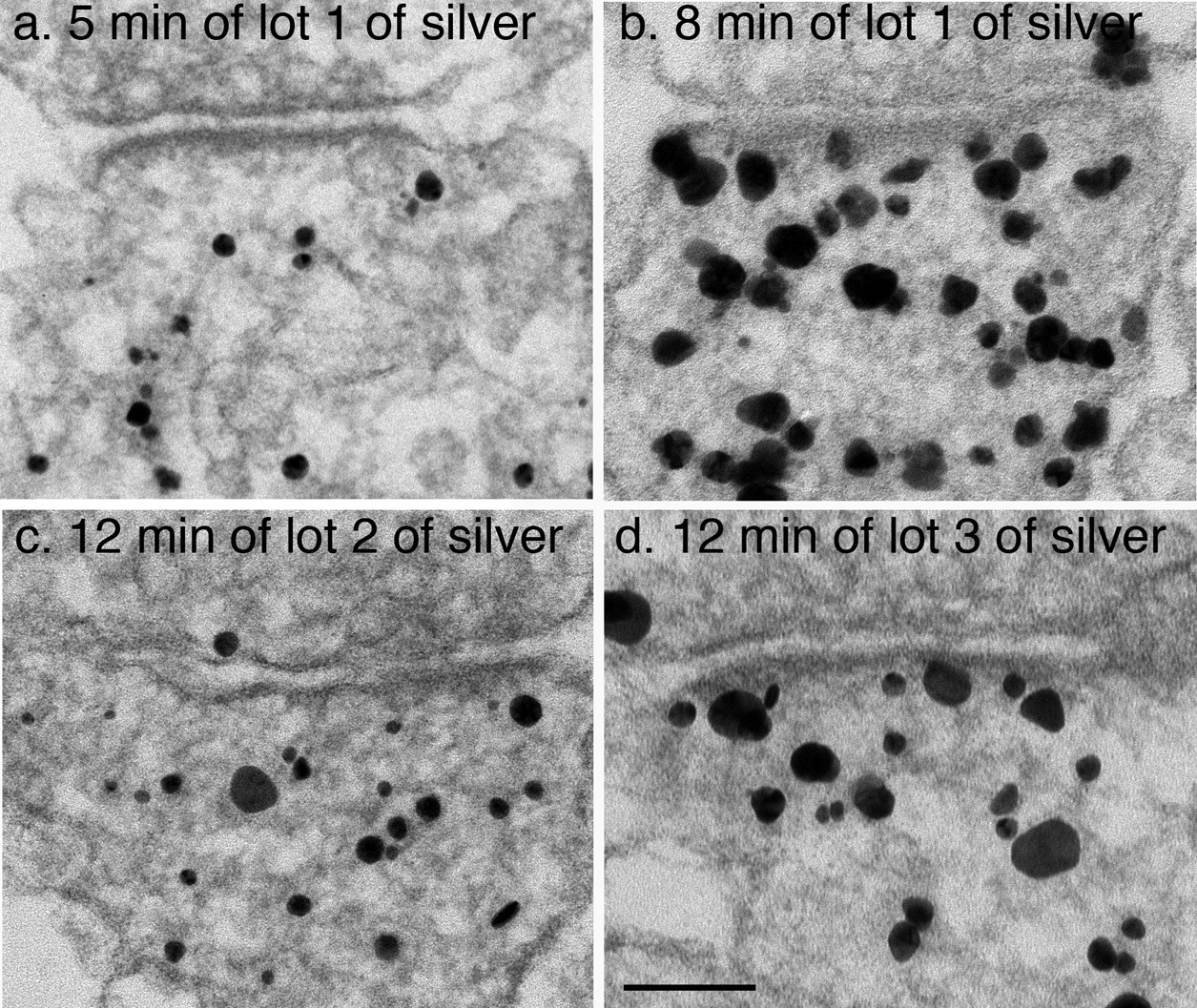


Synapses in dissociated hippocampal cultures labeled for CaMKII, and silver enhanced with different lots of Nanogold HQ kit. The sample enhanced for 5 min had smaller particles (a) than the parallel sample enhanced for 8 min (b). It should be noted that these samples were processed 15 years ago, and we have noticed that the HQ kits in recent years required longer development time (c & d). A recent pair of parallel samples was silver enhanced with two different lots of HQ kits for 12 min each. The sizes of the particles are smaller with one lot (c) than the other lot (d). Scale bar = 100 nm.
