# Supplementary material for: Optimization of protocols for pre-embedding immunogold electron microscopy of neurons in cell cultures and brains
Source: Mol Brain. 2021 Jun 3;14:86. doi: 10.1186/s13041-021-00799-2 (PMC8173732; doi:10.1186/s13041-021-00799-2)
Supplement: Supplementary file 8 — Additional file 8. Uranyl acetate en bloc treatment time affects labeling efficiency. [file 13041_2021_799_MOESM8_ESM.docx]

**Additional File 8. Uranyl acetate *en bloc* treatment time affects labeling efficiency.**


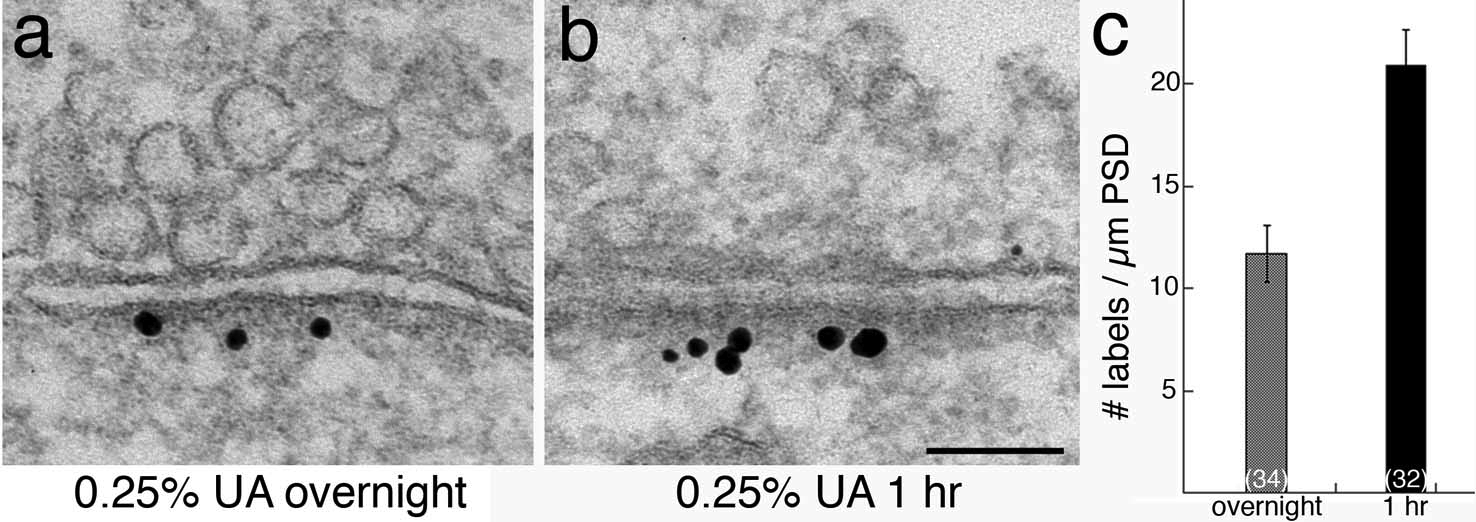


Dissociated hippocampal cultures were labeled with SynGAP, a Ras GTPase activating protein highly enriched in the PSD [29]. Parallel samples were treated with 0.25% uranyl acetate (UA) *en bloc* for overnight (a) or 1 hr (b). The overnight-treated sample had a higher contrast (a) but lower labeling density at the PSD (c). Scale bar = 100 nm. (c). Number of synaptic profiles measured listed at the bottom of the bar graphs (P<0.005 by Student’s t-test).
