# Supplementary material for: Optimization of protocols for pre-embedding immunogold electron microscopy of neurons in cell cultures and brains
Source: Mol Brain. 2021 Jun 3;14:86. doi: 10.1186/s13041-021-00799-2 (PMC8173732; doi:10.1186/s13041-021-00799-2)
Supplement: Supplementary file 9 — Additional file 9. Quality of silver particles in thin sections deteriorates over time. [file 13041_2021_799_MOESM9_ESM.docx]

**Additional File 9. Quality of silver particles in thin sections deteriorates over time.**


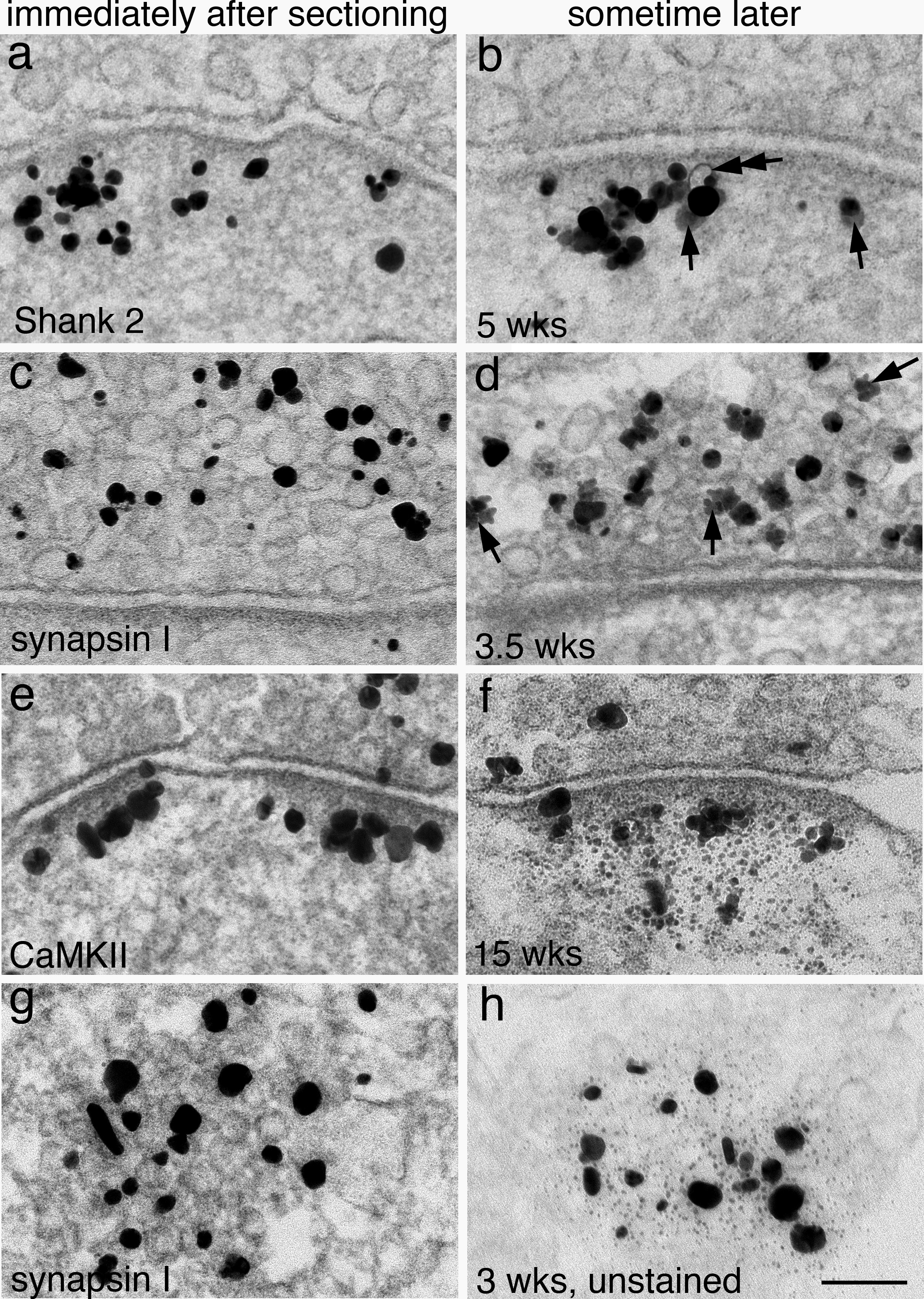


Sections from the same block of dissociated hippocampal culture examined immediately (left column) and later at different time points (right column): (a, b)- Sample was labeled with Shank 2, a PSD scaffold protein [21]. 5 wks later, some of the particles hollowed (double arrow in b) and some became grey (arrows in b). (c, d)- Sample was labeled with synapsin I, an SV-associated protein [18]. 3.5 wks later, some of the silver particles had irregular edges (arrows in d). (e, f)- Sample was labeled with CaMKII [6]. 15 wks later, silver particles became badly smudged (f). (g, h)- Sample was labeled with synapsin I. 3 wks later, the smudge of the silver particles was clearly visible in unstained sections (h), indicating that the smudging of the silver particles is not related to counterstaining. Scale bar = 100 nm.
